# Slow 0.1 Hz Breathing and Body Posture Induced Perturbations of RRI and Respiratory Signal Complexity and Cardiorespiratory Coupling

**DOI:** 10.3389/fphys.2020.00024

**Published:** 2020-02-14

**Authors:** Zoran Matić, Mirjana M. Platiša, Aleksandar Kalauzi, Tijana Bojić

**Affiliations:** ^1^Biomedical Engineering and Technology, University of Belgrade, Belgrade, Serbia; ^2^Faculty of Medicine, Institute of Biophysics, University of Belgrade, Belgrade, Serbia; ^3^Department for Life Sciences, Institute for Multidisciplinary Research, University of Belgrade, Belgrade, Serbia; ^4^Laboratory for Radiobiology and Molecular Genetics-080, Institute for Nuclear Sciences Vinča, University of Belgrade, Belgrade, Serbia

**Keywords:** complexity, RR interval variability, respiration rhythm variability, cardiorespiratory coupling, slow breathing, orthostasis

## Abstract

**Objective:** We explored the physiological background of the non-linear operating mode of cardiorespiratory oscillators as the fundamental question of cardiorespiratory homeodynamics and as a prerequisite for the understanding of neurocardiovascular diseases. We investigated 20 healthy human subjects for changes using electrocardiac RR interval (RRI) and respiratory signal (Resp) Detrended Fluctuation Analysis (DFA, α_1RRI_, α_2RRI_, α_1Resp_, α_2Resp_), Multiple Scaling Entropy (MSE_RRI1−4_, MSE_RRI5−10_, MSE_Resp1−4_, MSE_Resp5−10_), spectral coherence (Coh_RRI−Resp_), cross DFA (ρ_1_ and ρ_2_) and cross MSE (X_MSE1−4_ and X_MSE5−10_) indices in four physiological conditions: supine with spontaneous breathing, standing with spontaneous breathing, supine with 0.1 Hz breathing and standing with 0.1 Hz breathing.

**Main results:** Standing is primarily characterized by the change of RRI parameters, insensitivity to change with respiratory parameters, decrease of Coh_RRI−Resp_ and insensitivity to change of in ρ_1_, ρ_2_, X_MSE1−4_, and X_MSE5−10_. Slow breathing in supine position was characterized by the change of the linear and non-linear parameters of both signals, reflecting the dominant vagal RRI modulation and the impact of slow 0.1 Hz breathing on Resp parameters. Coh_RRI−Resp_ did not change with respect to supine position, while ρ_1_ increased. Slow breathing in standing reflected the qualitatively specific state of autonomic regulation with striking impact on both cardiac and respiratory parameters, with specific patterns of cardiorespiratory coupling.

**Significance:** Our results show that cardiac and respiratory short term and long term complexity parameters have different, state dependent patterns. Sympathovagal non-linear interactions are dependent on the pattern of their activation, having different scaling properties when individually activated with respect to the state of their joint activation. All investigated states induced a change of α_1_ vs. α_2_ relationship, which can be accurately expressed by the proposed measure—inter-fractal angle θ. Short scale (α_1_ vs. MSE_1−4_) and long scale (α_2_ vs. MSE_5−10_) complexity measures had reciprocal interrelation in standing with 0.1 Hz breathing, with specific cardiorespiratory coupling pattern (ρ_1_ vs. X_MSE1−4_). These results support the hypothesis of hierarchical organization of cardiorespiratory complexity mechanisms and their recruitment in ascendant manner with respect to the increase of behavioral challenge complexity. Specific and comprehensive cardiorespiratory regulation in standing with 0.1 Hz breathing suggests this state as the potentially most beneficial maneuver for cardiorespiratory conditioning.

## Introduction

The interaction of cardiac RRI and respiratory signal is a complex, mutually interrelated phenomenon. Related modern research poses questions like: why do RRI and respiratory signal values vary and what generates their complexity when forming a *meaningful, structural richness* (Grassberger, 1991)? Lack/decrease of RRI variability has been observed as a sign of pathology (Task Force Guidelines, 1996; Platiša and Gal, 2010; Valencia et al., 2013; Voss et al., 2013; Platiša et al., 2016a). Complementing homeostatic assumption, the lack of RRI oscillations (“oscillation death,” Stankovski et al., 2017) outlines a danger resulting from serious cardiac problems (Task Force Guidelines, 1996; Neves et al., 2012; Platiša et al., 2016b). Classical data on HRV refer to the changes of HRV in the linear domain, while more than 80% of HRV fluctuations belong to non-linear complex patterns (Yamamoto and Hughson, 1994). Although a few studies have pointed to increased complexity in the disease (Buccelletti et al., 2012; Valenza et al., 2017), it seems that the pathogenesis is most often followed by “de-complexification” (an increase of regular patterns in biological rhythm, Buccelletti et al., 2012; Sassi et al., 2015). So, complex and high rhythm variability refers to *homeodynamics* (Ernst, 2014) as a biophysical background of allometric physiological regulation (long term memory and multiscale correlations, West, 2010). Therefore, homeodynamics is a fundamental property of advanced biological sophistication.

Cardiac homeodynamics is a result of multilevel coupling: excitation-contraction coupling in the heart (Bers, 2018); hormonal regulation (Bai et al., 2009); thermoregulation (Fleisher et al., 1996); with autonomic nervous system (ANS) regulation as the dominant factor of this phenomenon. ANS regulation of cardiac homeodynamics is obtained by:

(i) sympathetic and parasympathetic effectors, with prevalently antagonistic, synchronous, synergetic, simultaneous (in-coupled) action on the heart (Zoccoli et al., 2001; Bojić, 2003, 2019; Silvani et al., 2003; Paton et al., 2005; Gierałtowski et al., 2013), and

(ii) coupling of cardiac rhythm with other biological oscillations, especially with the ones generated from breathing (i.e., central coupling of neural oscillators in ventrolateral medulla Porta et al., 2012; Schulz et al., 2013, 2018; Del Negro et al., 2018 and peripheral coupling dominated by the Bainbridge reflex (Bainbridge, 1930; Billman, 2011; Kapidžić et al., 2014).

Cardiopulmonary coupling is an intriguing phenomenon whose principal role, the energetic efficacy of oxygen transport, was recently found to extend to the adaptive capacity of the organism to internal and external challenges (Porges, 2007). This capacity for adaptation is investigated by the measurements of cardiopulmonary complexity by non-linear domain techniques (Goldberger, 2006). In the context of fundamental research, the majority of data on cardiovascular and respiratory autonomic patterns is based on the analysis of parameters of HRV linear domain. On the basis of these results, we deduce the antagonism of autonomic effectors on RRI regulation (change of posture, i.e., supine vs. standing, Montano et al., 1994; Levy and Martin, 1996; Jasson et al., 1997) or their synergism of action (i.e., supine vs. standing with slow breathing, de Paula Vidigal et al., 2016). These interrelated patterns of sympathetic vs. parasympathetic activity on RRI regulation are not confirmed for non-linear domain dynamics (Sassi et al., 2015).

Physiological states as RRI and respiration regulatory patterns include:

**Supine position (supin)**, considered the standard baseline for all cardiopulmonary physiological investigations. It is characterized by sympathetic withdrawal and small parasympathetic dominance on RRI regulation (Levy and Martin, 1996).

**Active standing (stand)**, a typical, well-characterized cardiocirculatory pattern of sympathetic dominance and vagal withdrawal on RRI regulation (Levy and Martin, 1996). The respiration pattern is characterized by increased ventilation and unchanged mean respiratory frequency with respect to supine position (Chang et al., 2005). With respect to supine position, this state is known for its beneficial effects on a number of neurocardiovascular (i.e., heart failure) and respiratory disturbances (Chang et al., 2004a,b; Zafiropoulos et al., 2004). The effect of active standing, to the best of our knowledge, has not been investigated with respect to the parameters of RRI, respiration and cardiopulmonary coupling in the non-linear domain, that could be of critical importance for the evaluation of RRI and respiratory adaptability on internal (i.e., disease state) or external (i.e., microgravity) challenges.

**Slow 0.1 Hz breathing**, a specific breathing frequency resulting from the maximum effect of respiration on RRI modulation (RSA, Eckberg, 1983; max Total Power of HRV, Cooke et al., 1998). This effect is vagally mediated and most probably obtained by system resonance effects of respiratory oscillatory drive on heart rate regulatory networks modulated by baroreflex (Julien, 2006; Castiglioni and Parati, 2011). This is, to the best of our knowledge, the maximal respiratory mediated physiological vagal drive on the heart. Its functional meaning was primarily attributed to energetic efficiency of the cardiorespiratory system, but also to the adaptability of the organism to unexpected environmental demands (Porges, 2007). Increased cardiorespiratory synchrony in slow 0.1 Hz breathing supports the energetic efficiency theory (Goldberger, 2006), but until now the question of cardiopulmonary adaptability was not addressed.

Specifically, regarding respiratory complexity, the change of posture and breathing regime are significantly interrelated with the breathing pattern (Mortola et al., 2016; Hernandez et al., 2019; Mortola, 2019). These two conditions, both individually and jointly, could give an insight into the contribution of (a) the peripheral factor for changed respiratory mechanics (horizontal vs. vertical plane) during orthostatic challenge, and (b) the impact of slow, voluntary 0.1 Hz control of breathing to the complexity regimes of the respiratory signal. Variability of the respiratory signal in the non-linear domain is of critical importance for the recovery of intensive care patients on artificial ventilation (Papaioannou et al., 2011). To the best of our knowledge, there are no data on the non-linear dynamics of respiratory signal in the conditions of peripheral respiratory drive change (change of posture) combined with the change of slow 0.1 Hz frequency respiratory drive. This interaction could be one of the critical mechanisms for the beneficial effect of posture change and slow breathing on critical care situations like weaning from artificial ventilation (Stiller, 2013).

Finally, as it goes for the simplest non-linear systems, RRI and respiratory regulation in coupled behavioral states like **supination with slow 0.1 Hz breathing** (**supin01**) and **standing with slow 0.1 Hz** breathing (**stand01**), most probably contravene the principles of proportionality and superposition (Goldberger, 2006). Slow 0.1 Hz breathing in two specific body postures could potentially have completely different effects on cardiorespiratory complexity parameters with respect to the predicted simple summation. Additionally, contrary to the previously investigated pharmacological joint *blockade* of sympathetic and parasympathetic activity on the RRI regulation (Silva et al., 2017a), to the best of our knowledge, cardiopulmonary complexity measures were not investigated in the state of joint physiological *enhancement/synergy* of sympathetic and vagal modulation of RRI (standing with slow 0.1 Hz breathing). This state was identified in the intensive care practice as the state of particular benefit for cardiopulmonary rehabilitation (Cooke et al., 1998; Bruton and Lewith, 2005; Dick et al., 2014; Russo et al., 2017).

### Cardiorespiratory Variables as an Insight Into Cardiorespiratory Cross Talk

Several studies have shown DFA exponent α to have a great power for probing complexity, as self-similarity across scale (Peng et al., 1995a,b, 2002; Ivanov et al., 1999; Fadel et al., 2004; Gierałtowski et al., 2013; Kristoufek, 2015; Barbiery et al., 2017). The advantages of fractal scaling exponents α_1_ and α_2_ over conventional methods like spectral analysis and Hurst exponent include the possibility of detecting long range correlations embedded in non-stationary/non-ergodic time series and of avoiding spurious detection of long range correlations that are the consequence of non-stationarities (Peng et al., 2002; Sassi et al., 2015). This method is validated (Peng et al., 1994) and successfully applied on both RRI (Peng et al., 1995a, 2002; Francis et al., 2002; Castiglioni et al., 2009, 2011) and respiratory interval time series (Peng et al., 2002; Fadel et al., 2004; Papaioannou et al., 2011). It quantifies information self-similarity across scale on both short term (α_1_) and long term time scales (α_2_).

MSE is another measure of signal complexity (i.e., irregularity) successfully applied on physiological signals (Costa et al., 2003) and in specific RRI (Silva et al., 2016, 2017a,b). It quantifies information irregularity (unpredictability) of sequence structural evolution in signal on both short term (MSE_1−4_) and long term time scales (MSE_5−10_).

Measures of self-similarity (DFA) and irregularity (MSE) are critical parameters of cardiovascular and respiratory system adaptability and physiologic plasticity (Goldberger, 2006). Fractal dynamics and irregularity in spontaneous RRI and respiratory signal fluctuations have implications for:

- Understanding physiological cardiopulmonary regulation
- Recognition of life-threatening cardiovascular events (i.e., heart failure—Silva et al., 2017a; Huikuri et al., 2000; Goldberger et al., 2002)
- Recognition of respiratory disturbances (i.e., adaptability of critically ill patients to spontaneous breathing—Papaioannou et al., 2011)
- Evaluation of detrimental effects of respiratory pathologies on neurocardiovascular physiology (Goulart et al., 2016). This ultimate factor unequivocally speaks in favor of the importance of understanding the cardiopulmonary coupling and its physiological background.

Finally, physiological non-linear signals like RRI (Peng et al., 1995a) and respiratory signal (Peng et al., 2002) couple (Moser et al., 2006; Schulz et al., 2018). The pattern and degree of the coupling can be evaluated both by means of linear and non-linear analytical methods (Podobnik and Stanley, 2008; Horvatic et al., 2011; Podobnik et al., 2011; Zebende, 2011; Blinowska and Zygierewicz, 2012; Kristoufek, 2014, 2015; Kwapien et al., 2015; Sassi et al., 2015). In accordance with that preposition, we applied spectral coherence (Coh_RRI−Resp_, in the linear domain), cross DFA and cross MSE (ρ and X_MSE_ in the non-linear domain, respectively) as the tools for estimating the level of cardiorespiratory coupling in four different physiological states. In order to investigate scale dependent changes of cardiopulmonary coupling of both complexity patterns, we separately analyzed cross DFA and cross MSE for short term and long term time scales (ρ_1_, ρ_2_ and X_MSE1−4_, X_MSE5−10_, respectively).

On the basis of the above facts we formulated the following working hypotheses:

- Individual posture changes and breathing regime changes differently affect RRI and respiratory complexity measures due to different mechanisms of regulation;
- Slow 0.1 Hz breathing could have posture dependent effect on RRI and respiration complexity measures;
- Standing with slow 0.1 Hz breathing could be regarded from the standpoint of cardiopulmonary complexity evaluation as a state of particular interest for cardiopulmonary adaptive conditioning;
- Different forms of cardiopulmonary coupling (Coh_RRI−Resp_, ρ, and X_MSE_) could have different, state-dependent patterns and these patterns could scale in dependent and mutually interrelated ways.

The scope of this comprehensive analysis was to analytically investigate complex state-specific synergetic and/or antagonistic patterns of RRI regulation, state-specific impact of body plane and breathing regime on respiratory regulation and to provide synthetic conclusions regarding the patterns of cardiopulmonary coupling. The four physiological states were chosen as typical patterns of RRI vegetative effectors' activity and respiratory regulation.

## Methods

### Subjects

We conducted the study protocol on 20 healthy adult human subjects (13 males, age 34.4 ± 7.4). The protocol was approved by the Ethical Committee of the Faculty of Medicine, University of Belgrade (No. 2650/IV-24). Criteria for inclusion of subjects into the study were: absence of any health problems and an age between 20 and 45 years. Exclusion criteria were: subjugation to any therapy (acupuncture, medications, etc.); a history of cardiovascular, pulmonar or any other diseases; presence of any health disorders at the time of the assessment or in the time leading up to the performance of the experimental measurements (such as cold, flu, pollen allergy, high temperature, migraines, etc.) and pathological symptoms during the experimental procedures (high blood pressure, arrhythmias, headache, fatigue, etc.). For female participants, an additional criterium of exclusion was the second part of menstrual cycle (because of its substantial and diverse cardiovascular autonomic regulation in females, Bai et al., 2009; Javorka et al., 2018). All participants were advised to refrain from food and drink from about 4 h before the experiment, not to exercise (running, gym, yoga, other), to be restful and alert.

Five participants (out of 25) were excluded because of pathological symptoms discovered during the recordings.

### Study Protocol

The study protocol was performed under controlled laboratory conditions at the Laboratory for Biosignals, Institute for Biophysics, Faculty of Medicine, University of Belgrade. It was conducted in a quiet, refreshing environment at a constant temperature (22 ± 1°C) during the experimental procedures for all subjects. Experiments were undertaken between 8 and 12 a.m., in order to control the circadian rhythm variability stemming from autonomic regulation (Bojić, 2003). All subjects were subjected to 10 min of relaxation in a supine position before recording. There was no restriction imposed on the air flow rate. Instead, subjects were advised to adjust the ventilation at the rate that felt most comfortable for them. They were also strictly instructed not to talk during the experimental procedures. The ECG (RRI) and respiration signals were simultaneously recorded in four conditions/sessions: supine and standing positions at spontaneous breathing rates, and in supine and standing positions with the slow paced 0.1 Hz breathing rates (supine, stand, supin01, and stand01, respectively). Session recordings lasted for 20 min, with a 5 min pause between the supine and standing position, in order to meet the criteria for cardiorespiratory complexity analysis (Peng et al., 1995a, 2002) and to obtain the stabilization of autonomic regulation in each state (Bojić, 2003). The sequence of these four sessions was randomly chosen, aiming at avoiding possible sequence influence on the experimental results. Slow breathing with a paced rhythm of 0.1 Hz was dictated by a computer web metronom sound^1^. Subjects adjusted each start of inhalation and exhalation according to the beap sound of the metronome. Thus, inhalation and exhalation in slow breathing sessions had equal durations. Subjects were trained and instructed for slow breathing regime before the recording sessions.

### Data Acqusition

ECG and respiration signal acquisition was done by means of Biopac MP100 system (Biopac System, Inc, Santa Barbara, CA, USA; AcqKnowledge 3.91 software). Main ECG lead registration electrodes were attached on the projections of clavicle bones and the grounding on the right ankle. The belt with resistive strain gauge transducer for continuous recording of breathing was placed slightly above the costal line. Both signals were sampled with 1,000 Hz frequency rate. We adjusted filters according to biopack instructions for general measurements: gain setting 10, low pass filter with 10 Hz and without high pass filter (DC-absolute respiratory measurement).

### Data Processing

We maintained controlled conditions during the recordings. Subjects were instructed to take a comfortable position which would allow them not to make any movements during the 20 min recording session. By visual analysis we agreed that there was no need for additional filtering of ECG signals. Respiration signal was low pass filtered (4th order Chebyshev filter) in order to erase little jitters physiologicaly appearing in the minimum level of expiration, but unrelated to research results (Kapidžić et al., 2014; Supplementary Data Sheet 1). The corresponding cut-off frequency was 1 Hz. RRIs were extracted from the ECG signal using Pick Peak tool in Origin (Microcal, Northampton, MA, USA; missed R peaks we added manually). Since the sample rate of the respiration signal was uniform (1,000 Hz), while RRI values form signals with unequally positioned samples (sampling frequency lower frequency than 1,000 Hz), a resampling of respiration signal was performed, according to the samples of RRIs. It was done using our custom Matlab program (Kapidžić et al., 2014; Supplementary Data Sheet 1).

The indices for our examination were: (a) linear measures of heart rate variability: mean value and standard deviation (Task Force Guidelines, 1996) (b) short term exponent α_1_ as a fractal measure which in heart rate strongly correlates with changes in low and high frequency oscillations (sympathetic and parasympathetic activity) (Weippert et al., 2015; Shiau, 2018); (c) long term exponent α_2_ as a fractal measure which in heart rate spectrum corresponds to a very low frequency band (Francis et al., 2002); (d) multiscaling entropy at short time scales (1–4 samples, MSE_1−4_), related to fast oscillations, respiratory and predominately vagal control (Silva et al., 2016); (e) multiscaling entropy at long time scales (5–10 samples, MSE_5−10_), related to slow oscillations, predominately of sympathetic control (Silva et al., 2016); (f) spectral coherence (Coh_RRI−Resp_), reflecting the presence (Daoud et al., 2018) and degree (Faes and Nollo, 2011) of linear cardiac and respiratory oscillatory synchronization; (g) short scale and long scale cross DFA (ρ_1_ and ρ_2_, respectively Podobnik and Stanley, 2008; Horvatic et al., 2011; Podobnik et al., 2011; Zebende, 2011; Kristoufek, 2015; Kwapien et al., 2015 as the parameters of cross correlations of fractal RRI and respiratory variations; and (h) short and long scale cross MSE (X_MSE1−4_ and X_MSE5−10_, respectively) as the measure of cross correlation in MSE domain (Costa et al., 2005). Programs for Cross DFA and cross MSE are available within Supplementary Data Sheet 1.

Non-linear indices of RRI and respiration were calculated using Matlab 2007b (Mathworks, Natick, USA). Applying an algorhithm for detrended fluctuation analysis, we obtained two numerical series: one with values of log (*F*(*n*)), the other for log (*n*). After ploting log (*F*(*n*)) vs. log (*n*), linear fit (regression line) was computed for the first 8 sample points (corresponding to *n* = 4–13). The slope of this regression line is regarded as the short term fractal scaling exponent α_1_. The same was done for the rest of the samples (following 16 points—*n* > 13), regarded as the long term fractal scaling exponent α_2_ (Peng et al., 1995a; Perakakis et al., 2009; please see Figure 4 in Appendix II). The number of points for short term α_1_ and long term α_2_ are not accidently chosen. They reflect two specific scaling regimes which are usually separated by a specific crossover point (discrete change of slope) in regression line (Peng et al., 1995b; Perakakis et al., 2009). In several subjects, the crossover was not positioned at the 9th point; for some subjects it was at an earlier point, such as the 6th, 7th, 8th, and for other subjects at a later point, such as the 10th and 11th point. Thus, in these cases we considered less points for obtaining α_1_ (5, 6, and 7 points, respectively) or later points for α_2_ (after 11th, 12th, etc). This occurred especially in sessions with slow breathing. Peng and co-workers noted that not all subjects exhibit crossover (and separation on two scaling regimes, Peng et al., 1995b), just as there were few cases of this kind in our sample. Characteristic crossover patterns are not just a feature of a healthy or diseased state, as Peng and co-workers pointed out (Peng et al., 1995b). Breathing frequency exerts influence on the crossover point as well (Perakakis et al., 2009; Platiša and Gal, 2010).

Moreover, we introduce here one additional measure, *inter-fractal angle* θ which reflects the relationship between two scaling regimes; in other words, it is an angle that short term and long term regression lines form between each other. In order to explain inter-fractal angle θ we conducted an angular analysis (detailed explanation in Appendix II). Instead of slopes of regression lines α_1_ and α_2_, angles that regression lines form with x-axis α_A1_ and α_A2_ were taken into account for the purpose of direct physical and physiological interpretation. Inter-fractal angle θ is directly proportional to the difference between α_A1_ and α_A2_ (θ = α_A1_ – α_A2_). We defined α_A1_ and α_A2_ as short term fractal angle and long term fractal angle with the abscissa, respectively. Additionally, our analytic tool characterizes the inter-fractal angle θ as a random variable, as well as its changes under the influence of orthostasis and slow breathing, which was analyzed using a probability density estimate procedure (PDE, supplied with Matlab, 2007b). In order to perform this analysis, the choice of inter-fractal angle θ with respect to the α_1_/α_2_ relation bypassed the possible calculation error for the case where slopes converge to infinite values (see Appendix II). Four numerical series (supine, stand, supin01, stand01), with 20 inter-fractal angle values each, were subjected to PDE analysis. Thus, we obtained four PDE profiles for four physiological conditions, in which distributions could be calculated (for detailed description please see Kalauzi et al., 2012). Additionally, we estimated PDE of the fractal angles α_A1_ and α_A2_. The aim of this was to try to elicit a physiological explanation of inter-fractal angle changes (please see Appendices II and III).

Multiscale entropies (MSE_1−4_ on short scales and MSE_5−10_ on long scales) were calculated as additional non-linear measures. They are based on the concept of sample entropy which by definition represents a “negative natural logarithm of the conditional probability that two sequences similar for *m* point intervals remain similar at the next point within a tolerance *r*” (Richman and Moorman, 2000). MSE algorithm makes estimation of sample entropy for each course-grained time series (averaged values from the data points within non-overlapping windows of increasing length/scale factor, Costa et al., 2005). Input criteria parameters for the sample entropy used had fixed values for all subjects: size of the window (pattern length) *m* = 2, and similarity criterion (standard deviation of a signal sequence) *r* = 0.15. The output of the algorithm consisted of two numerical series; one representing values of sample entropy for each scale factor and the other consisting of scale factor values (*n* = 1, …, 20). MSE_1−4_ was calculated as mean value from 1 to 4th sample points (sample entropy vs. scale factor), and MSE_5−10_ as mean value from 5 to 10th sample points (sample entropy vs. scale factor).

RRI-respiratory coherence (Coh_RRI−Resp_) was calculated using the following procedure: equidistant resampled RRI and respiration signals were imported in OriginPro 8.6 (OriginLab Corporation, Northampton, MA, USA). Within the Origin toolbox Analysis/Signal Processing/FFT/Coherence we made the following parameter settings: mean RRI for sampling interval of signals and Welch method for power spectral density estimation were chosen [decomposition of signal by Hanning window into smaller parts (256 points long), with 50% overlap (128 points)]. After the execution of the algorithm, two numerical rows were generated; one with values of frequency [Hz], the other with values of RRI-respiration cross power (variance) distributed over frequency ranges [s^2^/Hz]. Then, we plotted them as x vs. y coordinates, respectively, to get cross power spectrum as a function of frequency (see Figure 7 in Appendix IV). Using visual observation and peak pick tool, we determined the maximum value (peak) on the cross power spectrum diagram (Coh_RRI−Resp_). This usually corresponds with or near the location of breathing frequency (on the x-axis). We considered then that Coh_RRI−Resp_ represented the strength of the linear cardiorespiratory coupling. Values of Coh_RRI−Resp_ over 0.8 were assumed as high level/strong cardiorespiratory coupling. For a more detailed explanation of the application of the mentioned coherence method see Appendix IV (and/or Platiša et al., 2016a; Radovanović et al., 2018).

The short term and long term cross DFA (ρ_1_ and ρ_2_, respectively) parameters were calculated using the procedure described in Podobnik et al. (2011) and Kristoufek (2015) (see Supplementary Data Sheet 1). For every scale s, detrended cross-correlation coefficient was given by

$$\begin{array}{l} \rho_{DCCA}\left(s\right)=\frac{F_{DCCA}^{2}\left(s\right)}{F_{DFA,x}\left(s\right)F_{DFA,y}\left(s\right)} \end{array}$$

where $F_{DCCA}^{2}\left(s\right)$ is a detrended covariance between partial sums (profiles) of the two signals, while *F*_*DFA,x*_(*s*) and *F*_*DFA,y*_(*s*) are square roots of detrended variances of their partial sums. For each scaling range, both short (*s* = 4–13) and long (*s* = 14–108), this coefficient was averaged within the corresponding limits. Short term and long term scale cross MSE's (X_MSE1−4_ and X_MSE5−10_, respectively) were obtained by applying our custom made MATLAB program for calculating conventional cross sample entropy on signals previously prepared by coarse-graining procedure (Costa et al., 2005) (see Supplementary Data Sheet 1). For each scale range, these values were averaged (*n* = 1–4 for X_MSE1−4_ and *n* = 5–10 for X_MSE5−10_).

### Statistical Analysis

We stored all calculated results in a dataset crated with SPSS 19 (Statistical Package for the Social Sciences, 14, IBM, New York, USA). Statistical analysis was subsequently done by means of SPSS 19 toolboxes. We applied both visual checking of Gaussian distribution [by means of the frequency distributions (histograms), stem-and-leaf plot, boxplot, P-P plot (probability-probability plot) and Q-Q plot (quantile-quantile plot)] and Shapiro-Wilk normality test. Both visual checking and Shapiro-Wilk normality test of each parameter in 20 subjects confirmed that our data had non-Gaussian distribution. Therefore, we applied the non-parametric Kruskal Wallis test with *post-hoc* Mann Whitney test with Bonferroni's correction for multiple measurements to compare all samples (**Table 2**).

## Results

**Figure 1:**
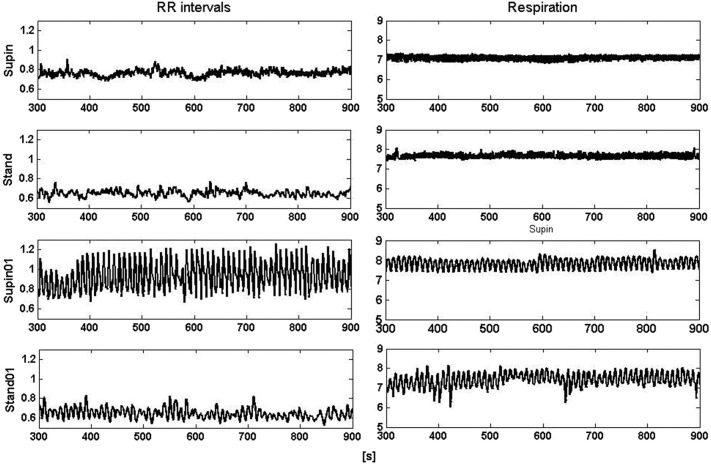
Segments (600 s) of RRI (left) and respiratory (right) signals recorded at supine position and standing with spontaneous (supin and stand, respectively) and 0.1 Hz breathing (supin01 and stand01, respectively).

It is obvious even from visual observation (Figure 1) that changes of body posture and breathing frequency affect RRI variability. While orthostasis causes a decrease in mean value and linear variability (standard deviation) of RRI, orthostasis with slow breathing results in the decrease of the RRI mean value only (Tables 1, 2). Supine position with slow breathing induced the highest values of mean linear RRI variability (sdRRI, Table 1).

**Table 1:** Linear and non-linear parameters (mean, SD) of 20 healthy subjects.

**Group**	**Parameter**	**Supin**	**Stand**	**Supin01**	**Stand01**
Cardiac parameters	mRRI [s]	0.9937 ± 0.1377	0.7263 ± 0.1021	1.0592 ± 0.1257	0.7480 ± 0.0867
	sdRRI [s]	0.0621 ± 0.0237	0.0465 ± 0.0175	0.0905 ± 0.0347	0.0702 ± 0.0225
	α_1RRI_	0.8975 ± 0.1925	1.3114 ± 0.1379	1.0342 ± 0.1421	1.3408 ± 0.1005
	α_2RRI_	0.8232 ± 0.1244	0.7874 ± 0.1249	0.6922 ± 0.1647	0.5545 ± 0.1463
	θ_RRI_ [^0^]	2.2 ± 8.3	14.5 ± 5.6	11.5 ± 8.7	24.6 ± 6.7
	α_A1RRI_ [^0^]	41.4 ± 5.9	52.5 ± 3	45.7 ± 4	53.2 ± 2.1
	α_A2RRI_ [^0^]	39.2 ± 4.4	38 ± 4.5	34.2 ± 6.7	28.6 ± 6.3
	MSE_RRI1−4_	1.7936 ± 0.1783	1.5583 ± 0.2974	1.6713 ± 0.2463	1.4715 ± 0.1784
	MSE_RR5−10_	1.7706 ± 0.2138	1.8951 ± 0.2391	1.4991 ± 0.1848	1.9123 ± 0.1732
Respiratory parameters	mResp [s]	4.55 ± 1.45	4.56 ± 1.78	10	10
	sdResp	0.89 ± 0.61	1.09 ± 1.35	0	0
	α_1Resp_	0.3679 ± 0.2603	0.4975 ± 0.2728	0.9268 ± 0.3133	1.1387 ± 0.2357
	α_2Resp_	0.5848 ± 0.2319	0.6119 ± 0.2132	0.4850 ± 0.2003	0.3759 ± 0.1028
	θ_Resp_ [^0^]	−10.3 ± 18.8	−5.5 ± 18.5	16 ± 16.1	27.5 ± 7.2
	α_A1Resp_ [^0^]	19.1 ± 11.4	25.2 ± 11.8	41.3 ± 12.1	47.9 ± 8.2
	α_A2Resp_ [^0^]	29.4 ± 10.6	30.7 ± 9.3	25.3 ± 10	20.4 ± 5.6
	MSE_Resp1−4_	1.4456 ± 0.2631	1.3185 ± 0.4117	1.3772 ± 0.3074	1.0995 ± 0.2837
	MSE_Resp5−10_	1.1396 ± 0.2532	1.0423 ± 0.3523	1.3040 ± 0.3065	1.3382 ± 0.3132
Cardio-pulmonary coupling	Coh_RRI−Resp_	0.8983 ± 0.0563	0.7397 ± 0.1986	0.8703 ± 0.1137	0.8663 ± 0.1363
	ρ_1_	−0.2419 ± 0.1905	−0.2002 ± 0.1916	−0.0096 ± 0.2665	−0.0697 ± 0.2787
	ρ_2_	−0.1346 ± 0.1314	−0.0190 ± 0.1234	−0.0232 ± 0.2471	0.0097 ± 0.2429
	X_MSE1−4_	2.2733 ± 0.20298	2.2719 ± 0.40199	2.1490 ± 0.24829	1.9344 ± 0.21773
	X_MSE5−10_	2.1765 ± 0.21385	2.1253 ± 0.27514	2.3176 ± 0.15034	2.4292 ± 0.46726

*Supin, supine position; stand, standing; supin01, supine position with paced 0.1 Hz breathing; stand01, standing with paced 0.1 Hz breathing; mRRI, mean value of RRI signal; sdRRI, standard deviation of RRI signal; α_1RRI_, short term fractal scaling exponent of RRI signal; α_2RRI_, long term fractal scaling exponent of RRI signal; θ_RRI_, inter-fractal angle of RRI signal; α_A1RRI_, short term fractal angle of RRI signal; α_A2RRI_, long term fractal angle of RRI signal; MSE_RRI1-4_, short term multiscaling entropy of RRI signal (for 1–4th sample); MSE_RRI5-10_, long term multiscaling entropy of RRI signal (for 5–10th sample); mResp, mean value of respiration signal; sdResp, standard deviation of respiration signal; α_1Resp_, short term fractal scaling exponent of respiration signal; α_2Resp_, long term fractal scaling exponent of respiration signal; θ_Resp_, inter-fractal angle of respiration signal; α_A1Resp_, short term fractal angle of respiration signal; α_A2Resp_, long term fractal angle of respiration signal; MSE_Resp1-4_, short term multiscaling entropy of respiration signal (for 1–4th sample); MSE_Resp5-10_, long term multiscaling entropy of respiration signal (for 5–10th sample); Coh_RRI−Resp_, RRI-respiration coherence; ρ_DCCARRI−Resp_, RRI-respiration detrended cross correlation coefficient; ρ_1_, short term scaling RRI-respiration detrended cross correlation coefficient; ρ_2_, long term scaling RRI-respiration detrended cross correlation coefficient; X_MSE1-4_, short term RRI-respiration cross multiscaling entropy; X_MSE5−10_, long term RRI-respiration cross multiscaling entropy*.

**Table 2:** Change of linear and non-linear cardiorespiratory parameters in different conditions.

**Group**	**Parameter**	**Supin-stand**	**Supin-supin01**	**Supin-stand01**
Cardiac parameters	mRRI	**0.001↓**	0.306	**0.001↑**
	sdRRI	**0.072↓**	**0.021↑**	0.831
	α_1RRI_	**0.001↑**	**0.030↑**	**0.001↑**
	α_2RRI_	>0.99	**0.027↓**	**0.001↓**
	θ_RRI_ [^0^]	**0.001↑**	**0.006↑**	**0.001↑**
	MSE_RRI1−4_	**0.015↓**	0.471	**0.001↓**
	MSE_RRI5−10_	0.120	**0.001↓**	0.063↑
Respiratory parameter	mResp	>0.99		–
	sdResp	>0.99		–
	$\alpha_{{1\text{Resp}}^{*}}$	0.273	**0.001↑**	**0.001↑**
	$\alpha_{{2\text{Resp}}^{*}}$	2.775	0.273	**0.001↓**
	θ_Resp_ [^0^]	0.942	**0.001↑**	**0.001↑**
	MSE_Resp1−4_	>0.99	>0.99	**0.001↓**
	MSE_Resp5−10_	>0.99	0.258	**0.054↑**
Cardio-pulmonary coupling	Coh_RRI−Resp_	**0.018↓**	>0.99	>0.99
	ρ_1_	1.194	**0.003↑**	**0.072↑**
	ρ_2_	0.015	0.228	0.105
	X_MSE1−4_	>0.99	0.402	**0.001↓**
	X_MSE5−10_	0.981	0.189	**0.051↑**

post-hoc Mann-Whitney test for independent samples with Bonferroni corrected p-value (p·m < 0.5, for m = 3, where m is the number of comparisons) after Kruskal-Wallis test for multiple comparation for 20 healthy subjects;↓-decrease of the change; ↑-increase of the change); supin-stand, supine position (with spontaneous breathing) vs. standing position (with spontaneous breathing); supine-supin01, supine position (with spontaneous breathing) vs. supination with paced 0.1 Hz breathing; supine-stan01, supine position (with spontaneous breathing) vs. standing with paced 0.1 Hz breathing; bolded numbers, results with statistical significance (p < 0.05); *Statistical significances of the respective angles were identical; mRRI, mean value of RRI signal; sdRRI, standard deviation of RRI signal; α_1RRI_, short term fractal scaling exponent of RRI signal; α_1Resp_, short term fractal scaling exponent of respiratory signal; α_2RRI_, long term fractal scaling exponent of RRI signal; α_2Resp_, long term fractal scaling exponent of respiratory signal; MSE_RRI1-4_, short term multiscaling entropy of RRI signal (for 1–4th sample); MSE_RRI5-10_, long term multiscaling entropy of RRI signal (for 5–10th sample); MSE_Resp1-4_, short term multiscaling entropy of respiratory signal (for 1–4th sample); MSE_Resp5-10_, long term multiscaling entropy of respiratory signal (for 5–10th sample); Coh_RRI−Resp_, RRI-respiration coherence; ρ_1_, short term scaling RRI-respiration detrended cross correlation coefficient; ρ_2_, long term scaling RRI-respiration detrended cross correlation coefficient X_MSE1-4_, short term RRI-respiration cross multiscaling entropy, X_MSE5-10_, long term RRI-respiration cross multiscaling entropy; grayshaded variables: variables which were not confirmed by Kruskal Wallis test as state dependent.

**Figure 2:**
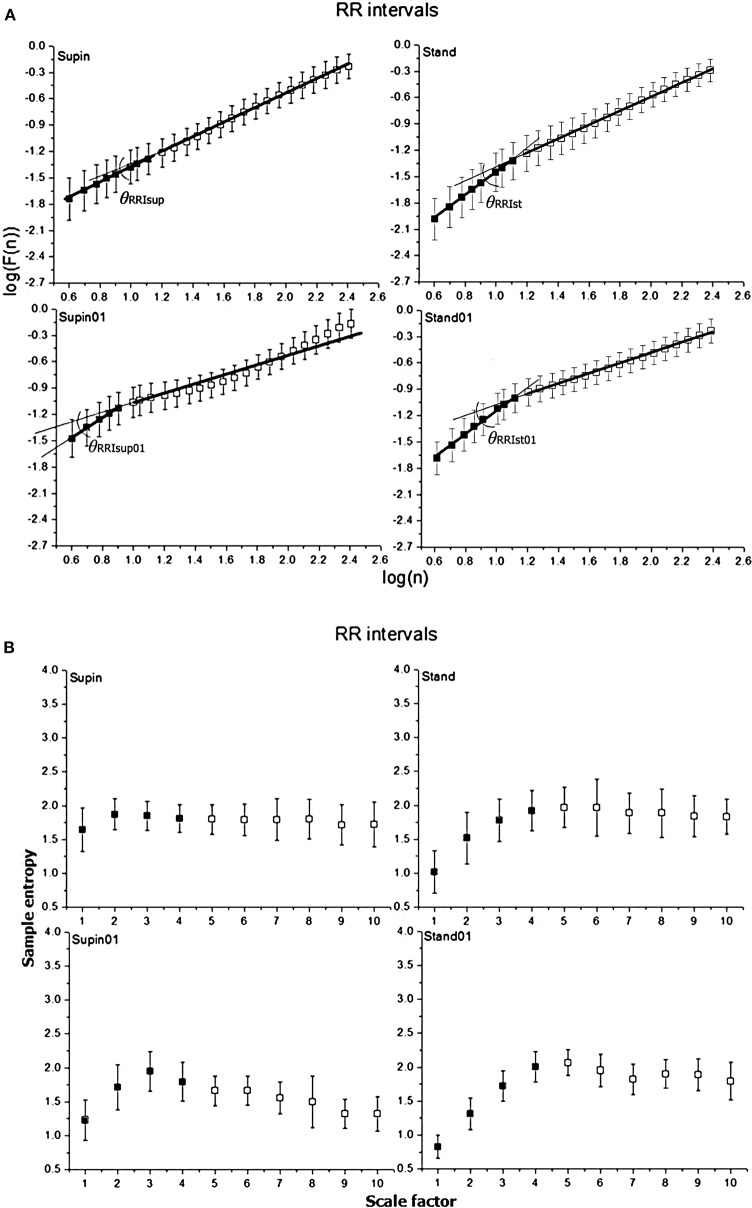
Graphic representation of non-linear properties of RRI variability in 20 healthy subjects expressed through: **(A)** fractal indices: full dark colored squares (dots) represent samples of short term fractal scaling exponent α_1_; empty squares represent samples for long term fractal scaling exponent α_2_; RRI inter-fractal angles: θ_RRIsup_, supine position with spontaneous breathing; θ_RRIst_, standing with spontaneous breathing; θ_RRIsup01_, supine position with paced 0.1 Hz breathing; θ_RRIst01_, standing with paced 0.1 Hz breathing; F(n), root-mean-square fluctuations, *n*, window size; **(B)** multiscaling entropy (1–20 samples); mean value of the first four samples (dark colored squares) is short term multiscaling entropy MSE_1−4_; mean value of 5–10th sample (light colored squares) is long term multiscaling entropy MSE_5−10_; supin, supine position; stand, standing; supin01, supine position with paced 0.1 Hz breathing; stand01, standing with paced 0.1 Hz breathing.

**Figure 3:**
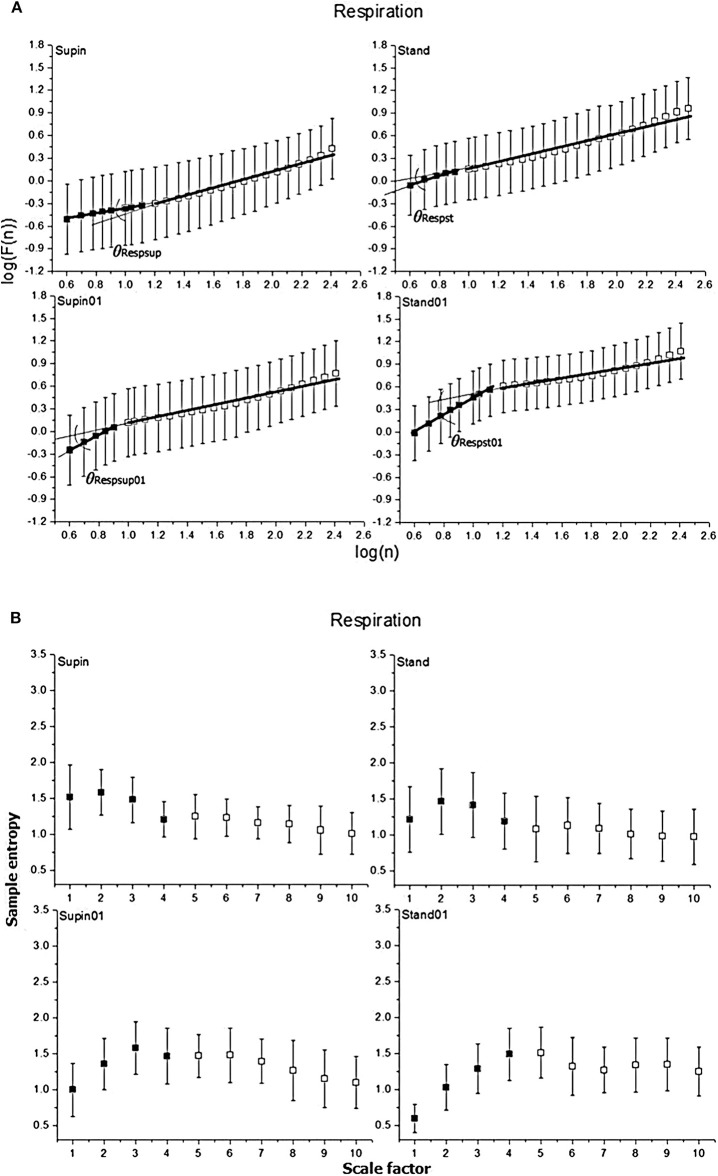
Graphic representation of non-linear properties of respiration signal in 20 healthy subjects expressed through: **(A)** fractal indices; full colored squares (dots) represent samples of short term fractal scaling exponent α_1_; empty (uncolored) squares represent samples for long term fractal scaling exponent α_2_; RRI inter-fractal angles: θ_RRIsup_, supine position with spontaneous breathing; θ_RRIst_, standing with spontaneous breathing; θ_RRIsup01_, supine position with paced 0.1 Hz breathing; θ_RRIst01_, standing with paced 0.1 Hz breathing; F(n), root-mean-square fluctuations, *n*, window size; **(B)** multiscaling entropy (1–20 samples); mean value of the first four samples (dark colored squares) is short term multiscaling entropy MSE_1−4_; mean value of 5–10th sample (light colored squares) is long term multiscaling entropy MSE_5−10_; supin, supine position; stand, standing; supin01, supine position with paced 0.1 Hz breathing; stand01, standing with paced 0.1 Hz breathing.

Mean values and standard deviations of non-linear parameters of RRI and respiratory signal variability are reported in Table 1. From the results calculated for 20 subjects, we calculated the horizontal mean value estimation in each sample of the non-linear parameter. Then, we plotted these mean values with their standard deviation as error bars (Figures 2, 3). On these plots we were able to observe changes of inter-fractal angle θ, a new quantity for y_1_ vs. y_2_ relationship, with superior accuracy with respect to the existing relations of slopes (De Souza et al., 2014, for details see Appendix II). Statistical significance of changes induced by body posture and breathing frequency on RRI and respiratory signal linear and non-linear parameters for 20 subjects are reported in Table 2.

State dependent changes of the coefficients are reported in Table 2. Due to the non-Gaussian distribution of the data confirmed by visual inspection and Shapiro-Wilk normality test, we applied the non-parametric Kruskal Wallis test. The variables that manifested significant state dependent change were compared with supine values (as the baseline) by Mann-Whitney test using the Bonferroni correction of the statistical significance from multiple permuted measurements (*p*·*m* < 0.5, for *m* = 3, where m is the number of comparisons^2^). The mean value of RRI (mRRI) was significantly changed just under the influence of orthostasis and the standard deviation of RRI (sdRRI) was significantly changed in supine with 0.1 Hz breathing. The short term scaling exponent α_1_ of RRI signal (α_1RRI_) was significantly increased under the influence of body posture (supin-stand), slow breathing (supin-supin01) and in the state of standing with 0.1 Hz breathing (supin-stand01). The long term scaling exponent α_2_ was significantly decreased in supine with slow breathing and in standing with slow breathing positions, while during orthostasis alone α_2_ was not significantly changed. The inter-fractal angle θ_RRI_ significantly increased in all three statistical conditions. This change was a consequence of the individual and joint change of α_A1RRI_ and α_A2RRI_ (Table 1, for detailed analysis see Appendix III). α_A1RRI_ increases both as a consequence of posture change (supin-stand) and a change of breathing regime (supin-supin01). α_A2RRI_ was decreased by slow breathing in two statistical cases (supin-supin01 and supin-stand01). Change of posture alone (supin-stand) did not result with a joint (opposite) change of α_A1RRI_ and α_A2RRI_, but by increase of α_A1RRI_ only.

Short term multiscaling entropy of RRI (MSE_RRI1−4_) was significantly decreased under the influence of body posture (supin-stand) and the change of body posture combined with the slow breathing regime (supin01-stand01). The long term multiscaling entropy (MSE_RRI5−10_) was increased by slow breathing in standing position (supin01-stand01, a significance level of *p* = 0.063), and decreased by slow breathing in supine position (supin-supin01). Joint (opposite) changes of MSE_RRI1−4_ and MSE_RRI5−10_ happened in the case of orthostasis with controlled breathing regime (supin01-stand01). The change of breathing regime (supin-supin01) only occurred when there was a change in MSE_RRI5−10_ (decrease). Of particular interest was the result that in stand01 both fractal (α_1RRI_ vs. α_2RRI_) and irregularity properties of RRI (MSE_RRI1−4_ vs. MSE_RRI5−10_) are reciprocally regulated. The analysis of scale dependent patterns revealed that both short scale (α_1RRI_ vs. MSE_RRI1−4_) and long scale (α_2RRI_ vs. MSE_RRI5−10_) parameters were also reciprocally regulated (Table 2).

In the respiratory signal, mean value and standard deviation (mResp and sdResp) changed only with the change of breathing regime (supin-supin01) and not with the change of posture (supin-stand). We also visually evaluated the respiratory signal DFA plot for the crossover point (Figure 3A) and applied the inter-fractal angle θ_Resp_ analysis analogous to the RRI signal analysis (Tables 1, 2). Detailed PDE analysis of the inter-fractal angle θ_Resp_ and its components are presented in Appendix III, Figure 6. α_1Resp_ did not change significantly with the posture change (supin-stand), but it increased in the case of controlled breathing regime (supin-supin01). α_2Resp_ did not change significantly either with the change of posture (supin-stand, *p* = 0.99) and in the condition of controlled breathing in supination (supin-supin01, *p* = 0.273). A significant decrease of α_2Resp_ was registered during the condition of standing with controlled breathing regime (supin-stand01). Joint changes of α_1Resp_ and α_2Resp_ were in the opposite direction. The inter-fractal angle θ_Resp_ did not change as a result of body posture change (supin-stand), but only under the controlled breathing regime (supin-supin01, supine-stand01, increase, *p* < 0.001).

The angle α_A1Resp_ did not change as the result of a body posture change (supin-stand), but significantly increased in all conditions with the controlled breathing regime (*p* < 0.001). The angle α_A2Resp_ also did not respond to the posture change (supine-stand) and slow breathing regime in supine position (supine-supin01, *p* = 0.273). α_A2Resp_ significantly decreased in the regime of slow breathing combined with standing (supin-stand01). Joint changes of α_A1Resp_ and α_A2Resp_ (supine-stand01) were in the opposite direction. State dependent, statistically confirmed changes of angles α_A1Resp_ and α_A2Resp_ were identical to the changes of the respective slopes (i.e., α_1Resp_and α_2Resp_; Table 2).

Change of α_1Resp_ (Δα_1Resp_, Table 3) was positive in all physiological conditions. Change of α_2Resp_ (Δα_2Resp_, Table 3) was negative only in conditions of slow 0.1 Hz breathing. Change of inter-fractal angle θ (Δθ_Resp_, see Table 3 and Appendix II) was always significant and positive in the conditions of controlled breathing regime (supin-supin01,), while insensitive to posture changes only (supin-stand).

**Table 3:** Change (arithmetic difference) of detrended fluctuation analysis parameters between physiological states.

**Parameter**	**Supin-stand**	**Supin-supin01**	**Stand-stand01**	**Supin01-stand01**
Δα_lRRI_	0.4139 ± 0.20127	0.1367 ± 0.15330	0.0294 ± 0.12612	0.3066 ± 0.16099
Δα_2RRI_	−0.0358 ± 0.16469	−0.1311 ± 0.20205	−0.2329 ± 0.12008	−0.1377 ± 0.19485
Δθ_RRI_ [^0^]	12.4 ± 10.3	9.3 ± 9.9	10 ± 4.4	13.1 ± 10.8
Δα_1Resp_	0.1296 ± 0.21130	0.5588 ± 0.36660	0.6412 ± 0.40181	0.2119 ± 0.39949
Δα_2Resp_	0.0271 ± 0.20977	−0.0998 ± 0.19600	−0.2360 ± 0.17368	−0.1091 ± 0.20976
Δθ_Resp_ [^0^]	4.8 ± 12.4	26.4 ± 20.3	32.9 ± 21.5	11.5 ± 20.8

*Supin, supine position; stand, standing; supin01, supine position with paced 0.1 Hz breathing; stand01, standing with paced 0.1 Hz breathing; Δα_1RRI_, change of short term fractal exponent α_1_ of RRI signal; Δα_2RRI_, change of long term fractal exponent α_2_ of RRI signal; Δθ_RRI_, change of inter-fractal angle of RRI signal; Δα_1Resp_, change of short term fractal exponent α_1_ of respiration signal; Δα_2Resp_, change of long term fractal exponent α_2_ of respiration signal; Δθ_Resp_, change of inter-fractal angle of respiration signal*.

Short term multiscaling entropy (MSE_Resp1−4_) was significantly decreased in conditions of combined standing position with slow breathing (supin-stand01). Long term multiscaling entropy (MSE_Resp5−10_) increased only in the condition of combined standing and slow breathing regime (supin-stand01, significance level of *p* = 0.054). In the condition of joint MSE_Resp1−4_ and MSE_Resp5−10_ change, the parameters changed in opposite directions.

We underline the result that in standing with 0.1 Hz breathing both fractal (α_1Resp_ vs. α_2Resp_) and irregularity properties of respiratory signal (MSE_Resp1−4_ vs. MSE_Resp5−10_) were reciprocally regulated. The analysis of scale dependent patterns revealed that in this state both short scale (α_1Resp_ vs. MSE_Resp1−4_) and long scale (α_2Resp_ vs. MSE_Resp5−10_) parameters were also reciprocally regulated (Table 2).

RRI-respiratory coherence (Coh_RRI−Resp_) was decreased under the influence of orthostasis (supin-stand). ρ_1_ significantly increased during slow breathing in supine position (*p* = 0.003) and standing with slow 0.1 Hz breathing (significance level of *p* = 0.072). Our statistical approach could not confirm state-dependent ρ_2_ changes. X_MSE1−4_ and X_MSE5−10_ decreased and increased, respectively, in the condition of orthostasis combined with slow breathing.

## Discussion

In recent years influence of slow breathing on heart rate variability (HRV) has been the focus or research (Russo et al., 2017). An increase of HRV has been recognized as one of the important physiological indicators of positive therapeutic effects of slow breathing techniques on the cardiovascular system (Bruton and Lewith, 2005; Dick et al., 2014; Russo et al., 2017) and the physiological indicator of cardiovagal function (Shields, 2009). Also, the research on the orthostasis effect on HRV has been well-documented (De Souza et al., 2014; Zaidi and Collins, 2016; Valente et al., 2018) and is routinely used as a sensitive test for the evaluation of “physiological adaptive mechanisms” generated by the autonomic nervous system (head up tilt, Zygmunt and Stanczyk, 2010; Hoshi et al., 2019). Most of the studies that evaluate HRV in these physiological conditions (supine position, standing, supine position with 0.1 Hz breathing and standing with 0.1 Hz) focused on linear properties of HRV (Kabir et al., 2011; de Paula Vidigal et al., 2016; Javorka et al., 2018; Jha et al., 2018). However, non-linear properties quantify and explain up to 80% of total RRI variability (Vandeput, 2010) and reflect physiological mechanisms of multiinteracting cardiovascular control, mostly exerted through sympatho-vagal effectors operating in non-linear fashion (de Godoy, 2016). Regarding the respiratory signal, a higher variability and complexity of respiratory rhythm was found in healthy subjects, while complexity decreases in the presence of diseases (Papaioannou et al., 2011; Reulecke et al., 2018). This is the first study which aimed to analyze parallel changes of RRI and respiratory rhythm complexity during individual and combined posture and breathing pattern changes. Ultimately the goal of this approach was to provide an insight into cardiorespiratory coupling in physiological conditions characterized by typical cardiac autonomic patterns, identifying the condition potentially most beneficial for cardiopulmonary adaptability.

As stated above, the actual evaluations of physiological RRI complexity measures were performed in the conditions of selective and total pharmacological *blockade* of sympathetic and parasympathetic system (Castiglioni et al., 2011; Silva et al., 2017a), posture change, mental stress (Castiglioni et al., 2009; Javorka et al., 2018), exercise and aging (Castiglioni et al., 2009). To the best of our knowledge, our approach is the first one to evaluate the physiologic background of RRI complexity measures in the conditions of physiological selective and joint *enhancement* of sympathetic (orthostasis) and parasympathetic (0.1 Hz breathing) activity on RRI regulation.

Changes of posture and slow 0.1 Hz breathing are also significantly interrelated with the breathing pattern (Mortola et al., 2016; Hernandez et al., 2019; Mortola, 2019), and, both individually and jointly, provide an insight into the contribution of (a) the peripheral factor of changed respiratory mechanics (horizontal vs. vertical plane, Mortola, 2019) during orthostatic challenge, and (b) the impact of central, slow 0.1 Hz breathing control on the complexity regimes of the respiratory signal (Papaioannou et al., 2011; Mortola et al., 2016; Reulecke et al., 2018). Finally, parallel evaluation of cardiorespiratory parameters and cardiorespiratory coupling by the RRI-Resp coherence, cross DFA and cross MSE provides an insight into cardiorespiratory integrative mechanisms in these conditions.

In order to verify the reproducibility of an autonomic pattern characteristic for supin, stand, supin01, and stand01 we calculated the following linear parameters: absolute values, changes of mean, and standard deviation of RRI. The absolute values and their changes were in accordance with the literature (Javorka et al., 2018; Valente et al., 2018), where supin was characterized by slight parasympathetic dominance (Levy and Martin, 1996), stand by sympathetic dominance (Table 1, decrease of mean RRI and SD with respect to supin, Table 2, Sobiech et al., 2017), supin01 with maximized parasympathetic dominance (Table 1, increase of SD with respect to supine, Shields, 2009) and stand01 with combined situation of higher sympathetic tone on mean RRI regulation (decrease of mRRI with respect to supin01, Table 1) with variability, most probably parasympathetically mediated, comparable to the supin values (Table 1, supin-stand01, Table 2, *p* = 0.831).

### α_1RRI_

A change of body posture (Table 1, supin-stand, sympathetic domination with parasympathetic withdrawal) determines a change in the α_1RRI_ parameter, from the value characteristic for the presence of long range correlations (supin, 0.5 < α_1RRI_ < 1, Peng et al., 1995a) toward Brownian noise (stand, α_1RRI_ → 1.5, Peng et al., 1995a). Changes of breathing pattern (Table 1, supin-supin01, parasympathetic domination) affect α_1RRI_, causing a shift from the value characteristic for long range correlations (supin, 0.5 < α_1RRI_ < 1, Peng et al., 1995a) toward 1/f noise (supin01, α_1RRI_ → 1, Peng et al., 1995a). Combined changes of body posture and slow breathing (Table 1, supin-stand01, α_1RRI_ → 1.5, Peng et al., 1995a) further increase α_1RRI_ toward Brownian noise. With respect to supin01, this change was even higher, shifting the quality of correlations from 1/f noise (Table 1, supin01, α_1RRI_ → 1, Peng et al., 1995a) toward Brownian noise (stand01, α_1RRI_ → 1.5, Peng et al., 1995a). The overall conclusion is that sympathovagal non-linear interactions might be dependent on the pattern of their activation, having different scaling properties when individually activated (i.e., sympathetic activation in stand, α_1RRI_ → 1.5, Brownian noise, vs. parasympathetic activation on supin01, α_1RRI_ < 1, 1/f noise, Table 2, *p* < 0.05 and *p* < 0.05, respectively) with respect to the state of their joint activation in stand01, where their non-linear RRI modulation appears to be additive, in the sense of Brownian noise (Table 2, *p* < 0.001).

### α_2RRI_

Change of body posture (Table 1, supin-stand, sympathetic domination with parasympathetic withdrawal) diminishes α_2RRI_ from the pattern of long range correlations (supin, 0.5 < α_2RRI_ < 1, Peng et al., 1995a) toward randomness (α_2RRI_ → 0.5, Peng et al., 1995a). This change was not significant (Table 2, *p* > 0.05). Change of α_2RRI_ by the change of breathing pattern (Table 1, supin-supin01, parasympathetic domination) significantly affects α_2RRI_ value toward that of a random pattern (Table 2). Combined changes of body posture with slow breathing (Table 1, stand-stand01) significantly decrease α_2RRI_ value toward randomness (Table 1, α_2RRI_ → 0.5, Peng et al., 1995a; Table 2, *p* < 0.05), both with respect to stand and with respect to supin01 (Table 1, α_2RRI_ → 0.5, supin01-stand01, Table 2, *p* < 0.05). The overall conclusion is that sympathetic and parasympathetic drive in the state of combined orthostasis and slow breathing (stand01) synergistically contribute to the increase of α_2RRI_ randomness, with greater contribution of parasympathetic drive with respect to sympathetic (Table 1, supin-supin01: *p* < 0.01; stand-stand01: *p* < 0.001, regarding the parasympathetic change, and supin-stand: *p* > 0.05; supin01-stand01: *p* > 0.05, regarding the sympathetic change).

### Δα_1RRI_

Change of α_1RRI_ was always positive in all body and breathing pattern changes with maximal change between supin-stand (Table 1, sympathetic dominance with parasympathetic withdrawal) and minimal change between stand-stand01, implying a potential additive effect of sympathetic activation and parasympathetic withdrawal on Δα_1RRI_ in the orthostasis on one side, and on the other side, potentially antagonistic action on Δα_1RRI_ of joint parasympathetic and sympathetic activation in stand01 condition (Table 3).

### Δα_2RRI_

Change of Δα_2RRI_ was always negative with minimum (absolute values) between supin-stand (sympathetic dominance with parasympathetic withdrawal) and maximum between supin01-stand01 (Table 3), implying a potential additive effect on Δα_2RRI_ of joint parasympathetic and sympathetic activation in stand01 condition.

These results of Δα_1RRI_ and Δα_2RRI_ (Table 3) imply that α_1RRI_ and α_2RRI_ are reciprocally regulated and mutually interdependent. This phenomenon was first noticed by Peng et al. (1995a) as a different α_1RRI_ vs. α_2RRI_ relationship between normal subjects and patients with congestive heart failure. This relationship was quantified as the α_1_/α_2_ ratio in physiological circumstances (women, change of posture, De Souza et al., 2014) but did not succeed in distinguishing state dependent RRI complexity changes. For this reason, we considered the angular values (θ, Appendices II and III), as more sensitive to individual and combined changes of the angle rays compared to the change of the α_1_/α_2_ index.

### θ_RRI_

In order to quantify the observed interdependence, we propose the inter-fractal angle θ_*RRI*_ between the linear regression lines of α_1RRI_ and α_2RRI_, with the vertex at the crossover point (Appendix II, Figures 2, 4). The angle θ_RRI_ has its minimal value in the supine position (Table 1, sympathetic withdrawal with slight domination of parasympathetic drive). θ_RRI_ significantly increased both with the change of body posture (Table 1, supin-stand) and breathing pattern (supin-supin01), with a maximum increase in a combined state (supin01-stand01). It is reasonable to deduce that individual and joint physiological enhancements of sympathetic and parasympathetic drive contribute to the increase of the inter-fractal angle θ_RRI_. We explored in detail the individual behaviors of α_A1RRI_, α_A2RRI_, and θ_RRI_ in four physiological conditions by PDE analysis (Appendix III). Figure 5A in Appendix III supports the view that supine state was characterized by multimodality of α_A1RRI_ generating regimes (three regimes, with dominant one approximately at mean 39°, with the greatest overall standard deviation). Change of posture shifted α_A1RRI_ toward unimodality (mean ~53° and decrease of overall standard deviation). Voluntary slow breathing induced lighter α_A1RRI_ regime homogenization with respect to the change of posture (shifting from trimodality to bimodality, with dominant regime on ~47° and with slightly decreased standard deviation). The most distinguished regime of α_A1RRI_ unimodality was in the circumstance of joint orthostasis with slow breathing (mean value of dominant regime 54°, the lowest value of standard deviation). α_A2RRI_ showed fewer characteristic changes, though bimodality could be observed both in orthostasis and slow breathing (Appendix III, Figure 5B) and the dominant regime in stand01 condition. A large θ_*RRI*_ standard deviation was characteristic of all four conditions. Inter-fractal angle θ_RRI_ reflected the PDE pattern and changes similar to α_A1RRI_ (trimodality in supin and the shift toward unimodality in stand, supin01 and stand01) with the most distinct unimodality in stand and stand01 conditions. These results are in accordance with the results of Castiglioni et al. (2009) that a basic physiologic, healthy regime (supin) was characterized by the spectrum of α_1RRI_ and α_2RRI_ coefficients, the non-linear variables analogous to the angles α_A1RRI_ and α_A2RRI_, as described by our analysis. To the best of our knowledge this is the first time that the spectrums of α_1RRI_ and α_2RRI_ are described by PDE and that the PDE pattern change was observed in four physiological conditions (supin, stand, supin01, stand01).

MSE_RRI1−4_ and MSE_RRI5−10_ also showed opposite changes in stand01 condition, suggesting that orthostasis with slow 0.1 Hz breathing was the determinant factor of this type of change. The pattern of joint MSE_RRI1−4_ and MSE_RRI5−10_ change was opposite to the pattern of joint α_1RRI_ and α_2RRI_ change (MSE_RRI1−4_ decrease and MSE_RRI5−10_ increase) indicating that these non-linear parameters do not reflect the same, but potentially complementary information on non-linear variability (Costa et al., 2003; Perakakis et al., 2009). Body posture reversed the direction of MSE_RRI5−10_ change induced by slow breathing (decrease for supin-supin01 and increase for supin-stand01, significance level of *p* = 0.063), which was suggestive of the hypothesis that the body posture might be the crucial factor for the direction of change of MSE_RRI5−10_. To the best of our knowledge, these are the first results on individual and joint effects of body posture and breathing regime on MSE_RRI1−4_ and MSE_RRI5−10_.

Regarding the respiratory signal, body posture did not change the linear parameters of breathing pattern (mResp and sdResp), while their change was obvious and expected with the change of breathing frequency. The same pattern, regarding the three statistical cases, was observed for mean values of all non-linear parameters (α_1Resp_, α_2Resp_, θ_Resp_, α_A1Resp_, α_A2Resp_, MSE_Resp1−4_, MSE_Resp5−10_; Table 2), implying that body posture change by itself cannot provoke the robust changes of mean values of non-linear respiratory parameters. This finding supports the opinion that mechanic changes (horizontal vs. vertical plane) and cardiocirculatory patterns specific for the posture state (supin-stand, Table 2) do not influence robustly the breathing pattern in the non-linear domain. Slow breathing in both statistical cases induced significant increases in θ_Resp_. In the supin-supin01 case, this increase was due only to the significant increase of α_1Resp_, while in supin-stand01 the change was obtained by the joint, opposite changes of α_1Resp_ (i.e., α_A1Resp_) and α_2Resp_ (i.e., α_A2Resp_) (Table 2). This result implies that short term (α_A1Resp_) and long term (α_A2Resp_) respiratory complexities are influenced in opposite directions by slow 0.1 Hz breathing coupled with a change in posture, making the change of θ_Resp_ more enhanced only with respect to the θ_Resp_ change by orthostasis (supin-stand; Table 2). This relationship between α_1Resp_ and α_2Resp_ (θ_Resp_) could hypothetically represent the result of confluent resonant cortical influences of posture maintenance motor system and slow 0.1 Hz respiration drive on brainstem autonomic respiratory network, considered to be an informational integrator of respiratory system (Feldman and McCrimmon, 2003).

PDE of α_A1Resp_ (Appendix III, Figure 6A) reveals two different bimodal distributions which changed the regime dominance pattern by posture change (from unidominant pattern in supin to equally represented bimodal regime in stand). Bimodality was significantly changed by slow 0.1 Hz breathing in the sense of shifting the dominant regime with the mean of ~11° to the regime with dominant regime at the mean of ~45°. The dominance of the unimodal pattern was even more enhanced by joint slow breathing in the standing position (mean α_A1Resp_ ~48°). α_A2Resp_ PDE (Appendix III, Figure 6B) was less sensitive on the posture change (multimodal regime pattern with low regime definition and high value of standard deviation). Slow breathing in the supine condition (supin01) defined two regimes of α_A2Resp_, with the dominant regime at the mean value of 28° and lower standard deviation with respect to the supine condition alone. Joint standing with slow breathing manifested clear regrouping of the two regimes into one, with mean of 22° and lower overall standard deviation. This data reveal that subtle, fine changes on breathing pattern in non-linear domain also happen during the postural change, but it appears that posture plays a role of secondary, enhancing factor of slow breathing impact on respiratory complexity. PDE analysis of inter-fractal angle θ_Resp_ (Appendix III, Figure 6C) illustrates the increase in multimodalities of the θ_Resp_ from prevailingly bimodal, with the dominant peak at −19° (supin), to potentially 5-modal regime in orthostasis (stand). Slow 0.1 Hz breathing introduced the shift of dominant pattern toward the regime of θ_Resp_ with mean of ~18° (supin vs. supin01). Standing with slow breathing induced dramatic regrouping of θ_Resp_ values into one dominant regime with a mean value of 26° and a low value of the standard deviation. The general conclusion is that the individual change of posture increases the number of modalities of all three angle parameters of respiratory complexity, while the individual slow breathing regime restricts this number. The maximal, apparent synergistic reductive effect on multimodalities of Resp angles was registered in the combined (stand01) state. This was in accordance with the fact that demanding posture requirements necessitate more adaptable respiratory patterns, also in non-linear domain, while cortical influences of slow breathing impose the inhibitory effect on the brainstem respiratory neural network chaotic properties and dictate a monomodal pattern of their non-linear operating mode. The state of stand01 could represent a qualitatively specific state, typical for the behavior of non-linear systems (Goldberger, 2006). Multimodality of Resp angles, only specific for the orthostasis in the function of respiratory adaptability to the diversity of expected environmental (i.e., behavioral) challenges, with one and only one imposed behavior (slow 0.1 Hz breathing), could become a qualitatively changed *enhancer* of 0.1 Hz breathing impact on Resp angles monomodal pattern.

MSE_Resp1−4_ and MSE_Resp5−10_ were parameters less sensitive to the change of breathing frequency, but were jointly modified in the condition supin-stand01.

Also in the case of respiratory signal complexity, in standing with 0.1 Hz breathing, both fractal (α_1Resp_ vs. α_2Resp_) and irregularity properties of respiration (MSE_Resp1−4_ vs. MSE_Resp5−10_) were reciprocally regulated. The analysis of scale dependent pattern revealed that in this state both short scale (α_1Resp_ vs. MSE_Resp1−4_) and long scale (α_2Resp_ vs. MSE_Resp5−10_) parameters were also reciprocally regulated (Table 2). Opposite fractal patterns were evident also for the state supin01 (α_1RRI_ increase, α_2RRI_ decrease, *p* < 0.05; α_1Resp_ increase, α_2Resp_ decrease, not significant), while this state was not characterized by the opposite change of the respective MSE scale pattern. This was also the case of the respective RRI parameters.

These results show that:

- The result of the scale dependent reciprocal pattern (α_1_ vs. MSE_1−4_) (α_2_ vs. MSE_5−10_) of both RRI and the respiratory signal in stand01 was not the consequence of calculation bias;
- Mechanisms responsible for the changes of self-similarity and irregularity properties of RRI and respiratory signal are independently regulated in the state supin01;
- The same RRI and respiratory complexity mechanisms are jointly and reciprocally regulated in the state stand01.

Cardiorespiratory regulation is integrated all along brainstem-hypothalamic axes up to limbic subcortical and cortical structures (Feldman and Ellenberger, 1988; Feldman and McCrimmon, 2003; Dampney, 2015). Behavioral control of breathing, with its specific voluntary component, is a state dependent, hierarchically organized dynamic system (Orem and Kubin, 2005; Kiselev and Karavaev, 2019; Noble and Hochman, 2019) with state dependent impact on cardiovascular regulation (best illustrated by the cardiovascular consequences of sleep apnea, Somers et al., 2008). These fundamental conclusions were drawn from the analysis of linear parameters of cardiorespiratory regulation.

The state specific pattern of both RRI and respiratory complexity regulation support the view that also RRI and respiratory complexity mechanisms are:

- Hierarchically regulated (loosely coordinated (“dual control,” Feldman and Ellenberger, 1988) cardiorespiratory control in individual behavioral tasks stand and supin01, transformed into well-defined and coordinated (“unitary control” Feldman and Ellenberger, 1988) cardiorespiratory response in the state of joined orthostasis with slow 0.1 Hz breathing).
- That hierarchical recruitment of regulatory complexity mechanisms most probably increases “bottom-up” with respect to the increment of the behavioral challenge (i.e., from medullar level toward higher diencephalo-telencephalic structures). The behaviorally most complex state in our experimental design, stand01, was characterized by reciprocal scale dependent and pattern specific cardiorespiratory response.

Regarding cardiopulmonary coupling, our data report for the first time that these linear and non-linear mechanisms are independently and differently engaged with respect to the behavioral state, where linear coupling (Coh_RRI−Resp_) appears to be sensitive on body posture change, while non-linear coupling (ρ_1_, X_MSE1−4_, and X_MSE5−10_) jointly and most dynamically change in the state of standing with 0.1 Hz breathing.

Cross DFA parameters ρ_1_ and ρ_2_ register anticross correlation, or 180° phase shift of RRI and respiratory signal in all four physiological states, with the exception of ρ_2_ in stand01 (Table 1). State dependent change was statistically confirmed only for ρ_1_ (Table 2). In the supine position, as the baseline state of reference, we registered maximal negative phase shift of RR and respiratory signal both for short and long scales. Minimal negative phase shift of RR and respiratory signal on short scales (ρ_1_) was noted in supin01 (Tables 1, 2, *p* = 0.003). This phenomenon was most probably the consequence of increased synchrony of RRI-Resp on short scales, due to the potentially maximal values of RSA in this condition.

Cross MSE parameters X_MSE1−4_ and X_MSE5−10_ report positive cross correlation in all four physiological states. Maximal degree of positive MSE cross correlation both for short and long scales was detected in supination, as the baseline state of reference. X_MSE1−4_ and X_MSE5−10_ were insensitive to individual posture and breathing pattern change, but jointly and oppositely changed in the condition of orthostasis combined with slow breathing (decrease and increase, respectively, Table 2) in the state of combined orthostasis and slow breathing. In that state ρ_1_ this manifests an increase of borderline significance (*p* = 0.072). A general conclusion might be that (a) ρ_1_, ρ_2_, X_MSE1−4_ and X_MSE5−10_ are not dependent on the body posture change; (b) cross DFA and cross MSE coupling regimes are most probably independently regulated, referring to different patterns of change with respect to the physiological state (supin01: ρ_1_ increase and X_MSE1−4_, X_MSE5−10_ not significant; stand01: ρ_1_ increase and X_MSE1−4_, X_MSE5−10_ decrease and increase, respectively). The results speak for the ρ_1_ positive correlation with the increase of vagal modulation to the heart, while X_MSE1−4_ and X_MSE5−10_ could correlate with synergic slow breathing and posture control.

Even though we are speaking about borderline significances (pρ_1_ = 0.072, pX_MSE5−10_ = 0.051) and solid statistical confirmation for X_MSE1−4_ (*p* < 0.0001), a general picture of state dependent changes of cardiopulmonary complexity identifies standing with slow 0.1 Hz breathing as the most composite but the best defined state. Regarding cardiopulmonary coupling, this state was characterized by a decrease of short scale irregularity coupling (**X**_MSE1−4_) and increase in short scale self-similarity coupling (**ρ_1_**). This opposite pattern of short scale cardiopulmonary coupling for ρ_1_ and X_MSE1−4_ was statistically confirmed only for the state of joint orthostasis with slow 0.1 Hz breathing, suggesting that only joint enhancement of volitional 0.1 Hz drive with sympathovagal modulation on the RRI could result in specific short scale coupling pattern. This cannot be attributed to vagal modulation only (traditional short scale RRI variability interpretation), but to the action of hierarchically higher structures on the sympatho-vagal pattern that potentiates short scale coupling in self-similarity and reduces short scale coupling in irregularity. The pattern of short scale cardiopulmonary coupling specific for the state stand01 could be a feedback information of particular importance for the higher order cardiopulmonary network (locus coeruleus, central nucleus of amygdala, paraventricular nucleus of hypothalamus, Noble and Hochman, 2019), dorsomedial hypothalamus and midbrain periaqueductal gray (Dampney, 2015). These structures are of essential importance for the organization of cardiopulmonary response to environmental threatening stimuli, i.e., cardiopulmonary adaptability to the challenges (Dampney, 2015). Long lasting stressful threats inevitably induce pathological plasticity changes at the functional level of integrative networks (Bajić et al., 2010; Dampney, 2015), and these changes are initially observed on the short scale feedback RRI regulatory processes (i.e., impairment of baroreflex function, Bajić et al., 2010; Park et al., 2017). Scale dependent change of cardiopulmonary coupling in different behavioral conditions has not investigated previously, to the best of our knowledge. Still, our results offer a solid basis for the hypothesis that, together with quiet sleep (Zoccoli et al., 2001), the state of combined standing with 0.1 Hz breathing could be (one of?) the state of short scale functional recovering process of the cardiopulmonary pathologic plasticity.

The role and the presence of long range components in this pattern of cardiopulmonary coupling could be followed by statistically discrete increases of MSE_RRI5−10_, MSE_Resp5−10_ and finally their increased coupling (X_MSE5−10_, *p* = 0.051). These results need further evaluation.

Finally, non-linear parameters of cardiorespiratory coupling had different patterns of state dependent change with respect to a linear effect; Coh_RRI−Resp_, suggesting that state dependent cardiopulmonary interaction is a multilevel, dynamically controlled phenomenon.

As a limited view, when speaking about cardiorespiratory coupling, we speak about mutual, bidirectional interaction between cardiac and respiratory oscillations (Porta et al., 2012; Dick et al., 2014; Radovanović et al., 2018). Besides neuro-humoral, there are also physical circumstances involved as a part of indirect cardiorespiratory coupling (Porta et al., 2012). Though it exerts small influence (Billman, 2011; Porta et al., 2012), it should not be completely underestimated. Bearing this in mind, multifactorial physical and neuro-humoral interplay contribute to state dependent heart-lung interrelations as a unique biophysical model of dynamic, coupled oscillators (Dick et al., 2014).

## Limitation of the Study

The ratio of spontaneous breathing inspirium vs. expirium duration (i/e) is ~1:2. In order to obtain sufficiently long RRI and respiratory signals for selected analysis and in physiological steady state of cardiorespiratory regulatory mechanisms, we designed 20 min registration sessions for each physiological state. Controlled 0.1 Hz breathing with i/e 1:2 was too fatiguing for examinees and we were compelled to apply the paced breathing in i/e relation 1:1.

The literature suggests that HF HRV and RSA are greater when breathing with a regime of low compared to high i/e ratio (Strauss-Blasche et al., 2000; Porges, 2007). In a study by Van Diest et al. (2014), where the influence of i/e relation during breathing frequency of 0.1 Hz (frequency of paced breathing) was specifically investigated, both 0.49 and 1.44 i/e ratio resulted in significant increase of RSA and decrease of HR, with respect to the baseline RSA and HR values for spontaneous breathing (Van Diest et al., 2014). This means that in both (extreme) situations of i/e relation we have parasympathetic dominance on HR regulation, the condition that we aimed to achieve. We consider useful to emphasize that our i/e condition (~1) during 0.1 Hz breathing is lower than the i/e condition of Van Diest et al. (2014) (1.44, an inverse relationship of i/e with respect to the value 1:2, typical for spontaneous breathing) and that consequently the difference between the parasympathetic drives to the heart of the two i/e conditions (0.49 vs. 1) could be negligible. Still, we recognize the potential limitation of this approach for the fine interpretation of respiratory mechanisms and we considered this caveat in the interpretation of the results.

## Conclusions

A major conclusion regarding parameters α_1RRI_ and α_2RRI_ is that they are reciprocally regulated and interdependent in four physiological conditions: supine, standing, supine with 0.1 Hz breathing and standing with 0.1 Hz breathing. That is in agreement with the existing literature (Peng et al., 1995a). This relationship can be described and quantified by the inter-fractal angle θ_RRI_, which was a sensitive parameter of the change of this relationship in investigated physiological states.

Regarding α_1RRI_, an orthostatic sympathetic increase contributes to α_1RRI_ in the sense of Brownian noise, while slow breathing parasympathetic increase contributes to the increase of α_1RRI_ in 1/f sense. In stand01 condition we report the maximal similarity of α_1RRI_ to Brownian noise, suggesting that physiological sympathovagal influence on short scale RRI self-similarity properties might be dependent on the pattern of their activation (i.e., individual vs. joint activation) and synergetic in the state stand01.

Regarding α_2RRI_, individual sympathetic and parasympathetic activation contribute to the increase of α_2RRI_ randomness, with greater contribution of parasympathetic drive with respect to sympathetic. In the state of combined orthostasis and slow breathing (stand01) this contribution appears synergetic.

PDE analysis of α_A1RRI_, α_A2RRI_, and θ_RRI_ revealed that baseline physiologic, healthy regime (supin) was characterized by the widest population (group) spectrum of α_1RRI_, α_2RRI_, and θ_RRI_ coefficients, which was in accordance with the results of (Castiglioni et al., 2009). PDE of these values is characterized by specific, state dependent changes of non-linear RR operating regimes. Again, the state of standing with 0.1 Hz breathing was the state of the best defined, maximal unimodality of all RRI angular parameters.

Additionally, in stand01 both fractal (α_1RRI_ vs. α_2RRI_) and irregularity properties of RRI (MSE_RRI1−4_ vs. MSE_RRI5−10_) are reciprocally regulated. The analysis of scale dependent patterns revealed that both short scale (α_1RRI_ vs. MSE_RRI1−4_) and long scale (α_2RRI_ vs. MSE_RRI5−10_) parameters were also reciprocally regulated (Table 2). All the results based on analysis of RRI complexity measurements speak in favor of stand01 being a qualitatively specific, regulatory well-defined state on multidimensional levels, where we reported the inter-relation of only two levels—horizontal (α_1RRI_ vs. α_2RRI_ and MSE_RRI1−4_ vs. MSE_RRI5−10_ relationships) and vertical (α_1RRI_ vs. MSE_RRI1−4_ and α_2RRI_ vs. MSE_RRI5−10_ relationships).

Non-linear parameters of respiratory signals (α_1Resp_, α_2Resp_, θ_Resp_, α_A1Resp_, α_A2Resp_, MSE_Resp1−4_, MSE_Resp5−10_) were robustly sensitive only to breathing regime change, while subtle PDE changes were observed as the result of the posture change. These changes were described mostly as a different number of operating regimes induced both by the change of posture and by the voluntary breathing regime. Demanding posture requirements necessitate more adaptable respiratory patterns, also in the non-linear domain, for the expected environmental (i.e., behavioral) challenges. Only one constant, long lasting and repetitive behavioral task, as was the slow 0.1 Hz breathing, qualitatively changed the feature of multimodality into a dominant monomodal respiratory pattern. Cortical influences of posture maintenance and slow breathing might jointly impose the inhibitory effect on brainstem respiratory neural network complexity properties and dictate monomodal pattern of their non-linear operating mode (Feldman and McCrimmon, 2003).

As a concluding remark, we stress that cardiorespiratory coupling in the non-linear domain is a highly dynamical, complex, interactive, state dependent phenomenon of cross talk between and within the cardiovascular and respiratory systems. This dynamical multilevel cross talk was also scale dependent, with different state dependent response patterns with respect to the patterns of changes in linear domain. The non-linear measures validating cardiopulmonary adaptability identify the state of standing with 0.1 Hz breathing as the most dynamic state, characterized by a specific complexity pattern, potentially beneficial for cardiopulmonary rehabilitation and conditioning. Future studies, on larger statistical samples, should address patterns of cardiopulmonary coupling in these and other states [i.e., exercise (Młynczak and Krysztofiak, 2018), sleep (Zoccoli et al., 2001), microgravity (Migeotte et al., 2003), neurocardiovascular pathologies Bojić, 2019] and potential parallel patterns of RR and respiratory variability changes both in linear and non-linear domain.

## Clinical Implications

One of the major implications of our research was the potential for cardiopulmonary rehabilitation. As we addressed in the Introduction, literature data report beneficial effects of slow 0.1 Hz breathing on cardiopulmonary rehabilitation. The opposite pattern of short scale cardiopulmonary coupling for **ρ_1_** and **X**_MSE1−4_, statistically confirmed only for the state of joint orthostasis with slow 0.1 Hz breathing, suggests that only joint enhancement of sympathetic and parasympathetic modulation on the RRI could result in the specific short scale coupling pattern. This pattern can be attributed to the resultant sympatho-vagal pattern that recruits and potentiates short scale cardiopulmonary coupling in self-similarity and reduces short scale coupling in irregularity. Since this was the first time that these results are reported, our statement is hypothetical and needs further evaluation.

Regarding the patients, if this state specific pattern of cardiopulmonary coupling was confirmed as the basis for the beneficial effect of slow breathing in orthostasis, this pattern could gain diagnostic value and become the scope of medical treatments by different approaches. Even though these phenomena were confirmed both for the respiratory system (“short term” and “long term facilitation,” Feldman and McCrimmon, 2003) and the cardiovascular system (Platiša et al., 2016b, 2019), a detailed description of the analog phenomena of cardiorespiratory interaction in healthy and patients needs to be addressed.

Finally, evaluation of cardiovascular and respiratory parameters of non-linear operational modes is of critical importance in intensive care unit patients. It was observed that low complexity of respiratory signal was a reliable prognostic sign of unsuccessful weaning of surgical critically ill patients from artificial ventilation (Papaioannou et al., 2011). Our data propose the evaluation of the rehab protocol for conscious artificially ventilated patients in the form of patient's slow voluntary breathing combined with orthostasis. On the basis of our results, hypothetically, this maneuver would potentiate the complexity of respiratory signal, promote the adaptive pattern of cardiopulmonary coupling and improve the odds for a successful weaning from artificial ventilation. This hypothesis necessitates clinical trials. Data obtained on integratory cardiorespiratory mechanisms might be of interest also for understanding the cardiorespiratory consequences of microgravity exposure (Migeotte et al., 2003; Prisk, 2014; Mandsager et al., 2015) and their successful surpassing by cardiorespiratory conditioning before and during the space flights.

## Data Availability Statement

The datasets generated for this study are available on request to the corresponding author.

## Ethics Statement

The studies involving human participants were reviewed and approved by Ethical Committee of Faculty of Medicine, University of Belgrade (No. 2650/IV-24). The patients/participants provided their written informed consent to participate in this study.

## Author Contributions

TB and MP designed the experimental protocol. TB, MP, and ZM recruited the research subjects. ZM performed the experiments and data acquisition. MP supervised the experiments. ZM performed the data analysis under MP, AK, and TB supervision. AK and MP programmed the algorithms. AK and ZM addressed the major computational tasks. ZM and TB did the scientific writing. TB gave the physiological interpretation of the data. All the authors approved the final content of the manuscript.

### Conflict of Interest

The authors declare that the research was conducted in the absence of any commercial or financial relationships that could be construed as a potential conflict of interest.

## Footnotes

^1^https://www.webmetronome.com/

^2^https://www.ibm.com/support/pages/post-hoc-comparisons-kruskal-wallis-test
