# Supplementary material for: Slow 0.1 Hz Breathing and Body Posture Induced Perturbations of RRI and Respiratory Signal Complexity and Cardiorespiratory Coupling
Source: Front Physiol. 2020 Feb 14;11:24. doi: 10.3389/fphys.2020.00024 (PMC7040454; doi:10.3389/fphys.2020.00024)
Supplement: Supplementary file 1 [file Data_Sheet_1.docx]

**Algorithms used in article Matić, Z., Platiša, M., Kalauzi, A., Bojić, T. “Slow 0.1 Hz Breathing and body posture induced perturbations of RRI and respiratory signal complexity and cardiorespiratory coupling”, Front. Physiol. doi: 10.3389/fphys.2020.00024**

**Abbreviations:**

**DFA** – detrended fluctuation analysis

**MSE** – multiscaling entropy

**CMSSEcor** – cross multi scaling sample entropy (corrected)

**FLP** – filtration low pass

**Upsampc** – upsampling c

**Upsampc68** – upsampling c 68 (if error occur in line 68)

**DCCA** – detrended cross correlation analysis

**DFA**

<https://www.physionet.org/physiotools/dfa/dfa.c>

**MSE**

<https://archive.physionet.org/physiotools/mse/mse.c>

**CMSSEcor**

load RRIsignal.txt

xpoc=RRIsignal;

nxpoc=length(xpoc);

load Respsignal.txt

ypoc=Respsignal;

nypoc=length(ypoc);

scmax=15

if ~exist('M')||isempty(M),M=2;end

if ~exist('r')||isempty(r),r=.15;end

if ~exist('sflag')||isempty(sflag),sflag=1;end

disp([' '])

for isc=1:scmax

disp(['scaling factor:' int2str(isc)])

if exist('x'),clear x;end

if exist('y'),clear y;end

for im=1:min(floor(nxpoc/isc),floor(nypoc/isc))

x(im)=mean(xpoc(isc*(im-1)+1:isc*im));

y(im)=mean(ypoc(isc*(im-1)+1:isc*im));

end

ny=length(y);

nx=length(x);

if sflag>0

y=y-mean(y);

sy=sqrt(mean(y.^2));

y=y/sy;

x=x-mean(x);

sx=sqrt(mean(x.^2));

x=x/sx;

end

A=zeros(M,1);

B=zeros(M,1);

for m=1:M

disp(['m=' int2str(m)])

pause(0.1)

for i=1:ny-m

for j=1:nx-m

if max(abs((x(j:j+m)-y(i:i+m))))<r

A(m)=A(m)+1;

end

end

end

A(m)=A(m)/((nx-m)*(ny-m));

%A(m)=A(m)/(nx-m);

for i=1:ny-m+1

for j=1:nx-m+1

if max(abs((x(j:j+m-1)-y(i:i+m-1))))<r

B(m)=B(m)+1;

end

end

end

B(m)=B(m)/((ny-m+1)*(nx-m+1));

end

p(:,isc)=A./B;

e(:,isc)=-log(p(:,isc))

end

**FLP**

ds=input(' file name. with samp. signal (without .mat): ','s');

nsamp=input('fr. samp. [samp/sec]:');

kp=input('begin. chanel. (1=<k=<nkan):');

kk=input('end. chanel. (1=<k=<nkan):');

if kk>kp+1

ki=input('chanel. exclude. (0 if all):');

else

ki=0;

end

fk=input('cutt of freq. for low pass [Hz]:');

nfil=input('insert order of filter (try with 4, or lower value):');

Rp=0.1;

Rs=9;

eval(['load ' ds])

eval(['[nodb,nkan]=size(' ds ');'])

nod=1;

nog=nodb;

eval(['dss=' ds '(nod:nog,:);'])

eval(['clear ' ds])

for k=kp:kk

xul=dss(:,k);

Wn=2*fk/nsamp;

[be,ae] = ellip(nfil,Rp,Rs,Wn);

[bc1,ac1] = cheby1(nfil,Rp,Wn);

xize(:,k)=filtfilt(be,ae,xul);

xizc1(:,k)=filtfilt(bc1,ac1,xul);

end

ksn=input('save on disc (d/n)?','s');

if ksn=='d'

dsout=input('enter name of file (without .mat):','s');

eval([dsout '=xizc1;'])

eval(['save ' dsout ' ' dsout])

disp(['file created:' dsout '.mat'])

end

**upsampc**

dsresp=input('name of input fale with filtrated rerspiratory signal (without .mat): ','s');

eval(['load ' dsresp])

eval(['resp=' dsresp ';'])

[nodbresp nkanresp]=size(resp);

t0=0.001;

t=t0:t0:nodbresp*t0;

respm=[t' resp];

dsrr=input('name of input file with corrected RR intervals (with .dat): ','s');

eval(['load ' dsrr])

eval(['absint=' dsrr(1:length(dsrr)-4) ';'])

[nodbrr nkanrr]=size(absint);

tup0=mean(absint(:,2));

tup=tup0:tup0:(nodbresp-1)*t0;

nodbup=min(length(tup),nodbrr);

figure

for iodbup=1:nodbup

iresp=floor(tup(iodbup)/t0);

x=tup(iodbup);

x1=respm(iresp,1);

x2=respm(iresp+1,1);

y1=respm(iresp,2);

y2=respm(iresp+1,2);

y=(y2-y1)/(x2-x1)*(x-x1)+y1;

respres(iodbup,:)=[x y];

end

plot(respres(:,1),respres(:,2))

hold on

for iodbup=1:nodbup-1

ispod=-1;

index=1;

while ispod==-1

ispod=sign(absint(index,1)-tup(iodbup));

index=index+1;

end

index=index-1;

**upsmpc68**

dsresp=input('name of input file with filtrated respiratory signal (without .mat): ','s');

eval(['load ' dsresp])

eval(['resp=' dsresp ';'])

[nodbresp nkanresp]=size(resp);

t0=0.001;

t=t0:t0:nodbresp*t0;

respm=[t' resp];

dsrr=input('name of input file with corrected RR intervals (with .dat): ','s');

eval(['load ' dsrr])

eval(['absint=' dsrr(1:length(dsrr)-4) ';'])

[nodbrr nkanrr]=size(absint);

tup0=mean(absint(:,2));

tup=tup0:tup0:(nodbresp-1)*t0;

nodbup=min(length(tup),nodbrr);

figure

for iodbup=1:nodbup

iresp=floor(tup(iodbup)/t0);

x=tup(iodbup);

x1=respm(iresp,1);

x2=respm(iresp+1,1);

y1=respm(iresp,2);

y2=respm(iresp+1,2);

y=(y2-y1)/(x2-x1)*(x-x1)+y1;

respres(iodbup,:)=[x y];

end

plot(respres(:,1),respres(:,2))

hold on

for iodbup=1:nodbup-1

ispod=-1;

index=2;

while ispod==-1

ispod=sign(absint(index,1)-tup(iodbup));

index=index+1;

end

index=index-1;

x=tup(iodbup);

x1=absint(index-1,1);

x2=absint(index,1);

y1=absint(index-1,2);

y2=absint(index,2);

y=(y2-y1)/(x2-x1)*(x-x1)+y1;

absintres(iodbup,:)=[x y];

end

plot(absintres(:,1),absintres(:,2),'r-o')

xlabel('time (s)')

legend('respir resampled','RR intervals')

title(dsresp)

title(dsresp)

ksn=input('save file with resampled respiratory and RRI signal (d/n):','s');

if ksn=='d'

eval(['save ' dsresp 'rup respres'])

disp(['formirana datoteka:' dsresp 'rup.mat'])

dsrsn=dsrr(1:length(dsrr)-4);

eval(['save ' dsrsn 'rup absintres'])

disp(['file created:' dsrsn 'rup.mat'])

end

**DCCA**

load RRIsignal.txt

load Respsignal.txt

Data1=RRIsignal;

Data2=Respsignal;

X=cumsum(Data1-mean(Data1));

Y=cumsum(Data2-mean(Data2));

X=transpose(X);

Y=transpose(Y);

N=length(X);

for n=[4 5 6 7 8 10 11 13 16 19 23 27 32 38 45 54 64 76 91 108]

for i=1:N-n

ind1=i;

ind2=i+n-1;

index=(ind1:ind2);

C0=polyfit(index,X(index),1);

C1=polyfit(index,Y(index),1);

fit0=polyval(C0,index);

fit1=polyval(C1,index);

a=1/(n-1);

CovXY(i)=a*sum((X(index)-fit0).*(Y(index)-fit1));

CovX(i)=a*sum((X(index)-fit0).^2);

CovY(i)=a*sum((Y(index)-fit1).^2);

end

CovXYm(n)=mean(CovXY);

VarX(n)=mean(CovX);

VarY(n)=mean(CovY);

ro(n)=CovXYm(n)/(sqrt(VarX(n))*sqrt(VarY(n)));

end

ro
