# Supplementary material for: Slow 0.1 Hz Breathing and Body Posture Induced Perturbations of RRI and Respiratory Signal Complexity and Cardiorespiratory Coupling
Source: Front Physiol. 2020 Feb 14;11:24. doi: 10.3389/fphys.2020.00024 (PMC7040454; doi:10.3389/fphys.2020.00024)
Supplement: Supplementary file 2 [file Data_Sheet_2.docx]

**Appendices**

Appendix I

As introduced in ref (Peng et al., 1995a) detrended fluctuation analysis (DFA) was used for obtaining fractal measure of correlation properties in random like time series. DFA algorithm applied for our RRI signals comprise:

- integration of RRI time series: $y\left( k \right)=\sum_{i=1}^{k} \left[ \mathrm{RRI}_{i}-\mathrm{RRI}_{\mathrm{mean}} \right]$, where RRI(i) was the ith R-R interval and RRI_mean_ was the mean RRI;


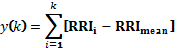

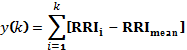


- dividing into sequences of equal length, n;

- a least-squares line fitting through data points (linear trend) in each of the sequences;

- detrending the integrated time series y(k) by subtracting the local trend (y-coordinate of the straight line) y_n_ (k), in each sequence;

- calculation of the characteristic size of fluctuation for integrated and detrended time series with sequence length n by formula: $F\left( n \right)=\sqrt{\frac{1}{N}\sum_{i=1}^{k} \left[ y\left( k \right)-y_{n}\left( k \right) \right]^{2}}$;


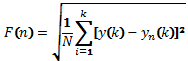


-repeating calculation of F(n) for all different lengths/time scales (n) which enables functional relationship F(n) and n in logarithmic axis scales (Figure 4).

Completely analogous procedure of DFA was applied for respiration signal.

Appendix II

**Basic mathematics behind angular analysis in DFA inter-relatedness**

If we observe one example of results estimated by detrended fluctuation analysis (DFA), geometrical relatedness was clearly visible (Figure 4). In references on application of DFA, instead of angles intuitive for evaluation of results, slopes of regression lines (α_1_ and α_2_) are usually taken into account.

The relation between the slope and the angle of the line is that slope is “tangens” function of the angle. Tangens is not linear function of the angle. It is approximately linear function of the angle only for small values of the angle, while, as it approaches to the value of 90 degrees, tangens function trends towards infinite values. Even though we do not deal with the angles close to 90 degrees, with regard to this deformation it is more precise to deal with angles than the slopes. This measure made possible the Probability Density Estimate analysis (Appendix III) which revealed that not only tangens of individual slopes (α_1_ and α_2_, Castiglioni 2009), but also their differences exhibited a spectrum of values, which were state specific.

Finally, the relation of α_1RRI_/ α_2RRI_ was shown as insensible to the posture changes in healthy women (De Souza et al., 2014), while the proposed angle *θ_RRI_* gave state dependent changes for all three statistical cases.

So, observing an example of DFA results, we can notice few specific angles.

**
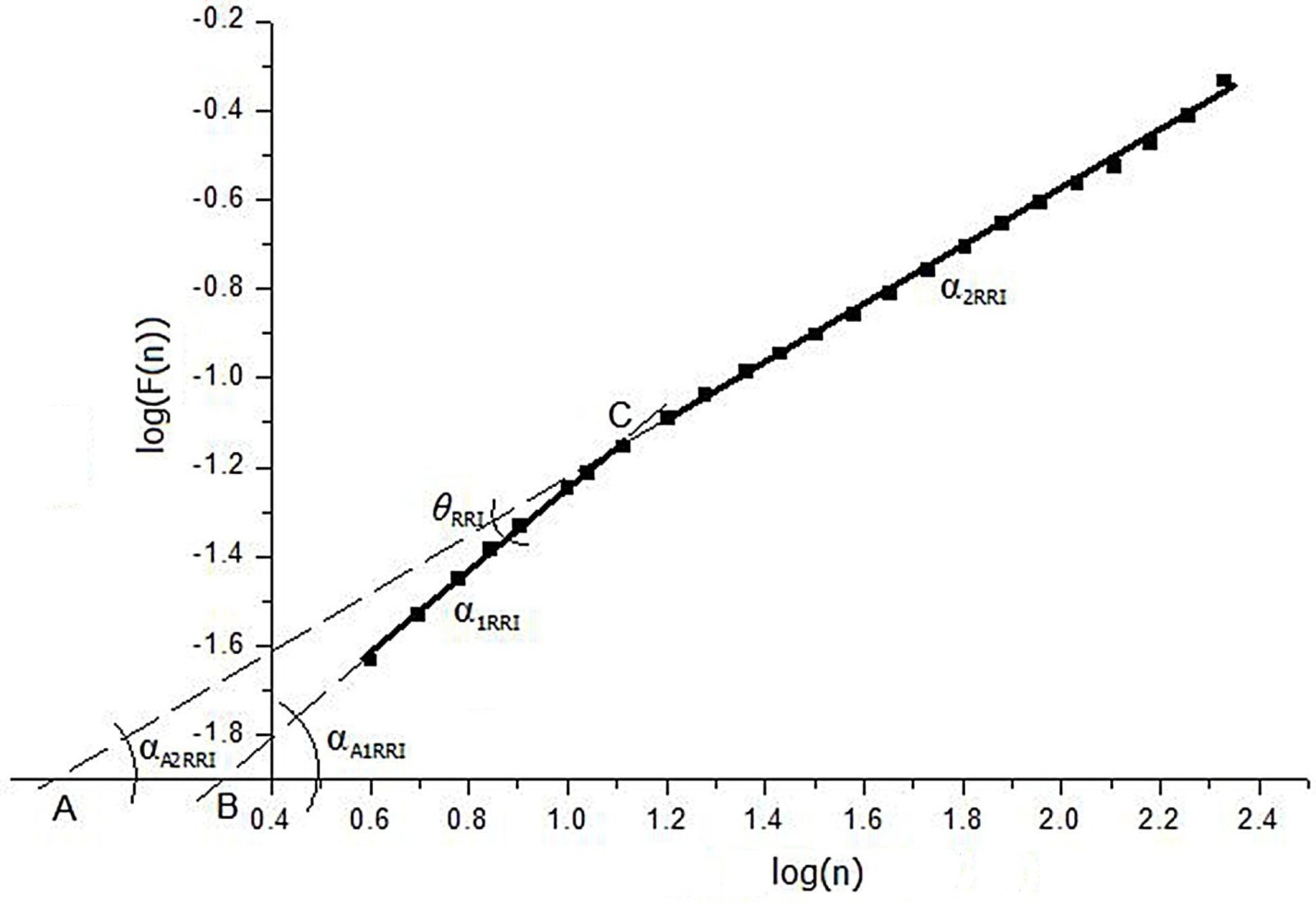
**

**Figure 4.** Angles of regression lines on detrended fluctuation analysis (DFA) diagram obtained from RRI signal of a representative subject during supination with spontaneous breathing; θ_RRI_ – inter-fractal angle (angle that regression lines form between each other); α_1ARRI_ – short term fractal angle (angle that regression line y_1_ form with X-axis); α_2ARRI_ – long term fractal angle (angle that regression line y_2_ form with X-axis); α_1RRI_ – short term fractal scaling exponent (slope of regression line y_1_); α_2RRI_ – long term fractal scaling exponent (slope of regression line y_2_); A – point of interception with X-axis made by y_2_ regression line, B – point of interception with X-axis made by y_1_ regression line; C – cross over point between y_1_ and y_2_ regression lines.

As mentioned, we are especially considering angle that regression lines y_1_ and y_2_ (α_1_ and α_2_ fractal regimes) are forming between each other. We calculated it according to simple derivation:

$y_{1}=A_{1}+\alpha_{1\mathrm{RRI}}X_{1}$ – equation of regression line (linear fit) from short term samples on log(F(n)) vs log(n) diagram; where α_1_ represents the slope of the line (short term scaling exponent).


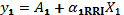

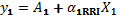


$y_{2}{=A}_{2}+\alpha_{2RRI}X_{2}$ – equation of regresion line (linear fit) from long term samples on log(F(n)) vs log(n) diagram; where α_2_ represents the slope of the line (long term scaling exponent).


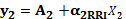

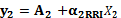


$\alpha_{A1\mathrm{RRI}}=\mathbf{∡}\mathrm{CB}X=\arctan\alpha_{1\mathrm{RRI}}\cdot\frac{180}{\pi}$ - fractal angle that y_1_ forms in respect to x-axis.


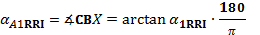

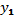


$\alpha_{A2RRI}=∡CAX=\arctan\alpha_{2RRI}\cdot\frac{180}{\pi}$ - fractal angle that y_2_ forms in respect to x-axis.


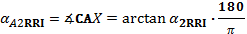


Inter-fractal angle *θ* between regression lines y_1_ and y_2_ could be obtained from triangle ABC:

$$\alpha_{A1\mathrm{RRI}}^{'}=\mathbf{∡}CBA={180}^{0}-\alpha_{A1\mathrm{RRI}}$$

$$\alpha_{A1\mathrm{RRI}}^{'}+\alpha_{A2\mathrm{RRI}}+\theta_{\mathrm{RRI}}={180}^{0}$$

$\theta_{\mathrm{RRI}}={180}^{0}-\alpha_{A1\mathrm{RRI}}^{'}-\alpha_{A2\mathrm{RRI}}$ , where

$$\theta_{\mathrm{RRI}}={180}^{0}-{180}^{0}+\alpha_{A1\mathrm{RRI}}-\alpha_{A2\mathrm{RRI}}$$

$$\theta_{\mathrm{RRI}}=\alpha_{A1\mathrm{RRI}}-\alpha_{A2\mathrm{RRI}}$$

Since we have already calculated α_1RRI_ and α_2RRI_, it is possible to derive simple expression for *θ*:


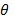


$\tan\theta=\tan\left( \alpha_{A1\mathrm{RRI}}-\alpha_{A2\mathrm{RRI}} \right)$

$$\tan\theta_{\mathrm{RRI}}=\frac{\tan\alpha_{A1\mathrm{RRI}}-\tan\alpha_{A2\mathrm{RRI}}}{1+\tan\alpha_{A1\mathrm{RRI}}\cdot\tan\alpha_{A2\mathrm{RRI}}}$$

$$\tan\theta_{\mathrm{RRI}}=\frac{\alpha_{1\mathrm{RRI}}-\alpha_{2\mathrm{RRI}}}{1+\alpha_{1\mathrm{RRI}}\cdot\alpha_{2\mathrm{RRI}}}$$

$$\theta_{\mathrm{RRI}}=\arctan\left( \frac{\alpha_{1\mathrm{RRI}}-\alpha_{2\mathrm{RRI}}}{1+\alpha_{1\mathrm{RRI}}\cdot\alpha_{2\mathrm{RRI}}} \right)\cdot\frac{180}{\pi}$$

This general formula was applied for each estimation of angles in all four sessions/conditions for RRI signal. Applied for supination and standing in RRI signal it gets specified form:

$$\theta_{\mathrm{RRIsup}}=\arctan\left( \frac{\alpha_{1\mathrm{RRIsup}}-\alpha_{2\mathrm{RRIsup}}}{1+\alpha_{1\mathrm{RRIsup}}\cdot\alpha_{2\mathrm{RRIsup}}} \right)\cdot\frac{{180}^{0}}{\pi}$$

$$\theta_{\mathrm{RRIstand}}=\arctan\left( \frac{\alpha_{1\mathrm{RRIstand}}-\alpha_{2\mathrm{RRIstand}}}{1+\alpha_{1\mathrm{RRIstand}}\cdot\alpha_{2\mathrm{RRIstand}}} \right)\cdot\frac{{180}^{0}}{\pi}$$

Analogous specified formulas were used for angles in supination with slow breathing (supin01) *θ*_RRISup01_, standing with slow breathing (stand01) *θ*_RRIStand01_ in RRI signal. Analog principle was applied on DFA regression lines for respiratory signal: in supination *θ*_RespSup_, standing *θ*_RespStand_, supination with slow (0.1 Hz) breathing *θ*_RespSup01_ and standing with (0.1 Hz) slow breathing *θ*_RespStand01_.

Change of angle under the influence of orthostasis (the difference between supination and standing) in RRI signal (*Δθ*_RRIsupstand_) was calculated as:

$$\Delta\theta_{\mathrm{RRIsupstand}}=\theta_{\mathrm{RRIsup}}-\theta_{\mathrm{RRIstand}}$$

$$\Delta\theta_{\mathrm{RRIsupstand}}=\arctan\left( \frac{\alpha_{1\mathrm{RRIsup}}-\alpha_{2\mathrm{RRIsup}}}{1+\alpha_{1\mathrm{RRIsup}}\cdot\alpha_{2\mathrm{RRIsup}}} \right)\cdot\frac{{180}^{0}}{\pi}-\arctan\left( \frac{\alpha_{1\mathrm{RRIstand}}-\alpha_{2\mathrm{RRIstand}}}{1+\alpha_{1\mathrm{RRIstand}}\cdot\alpha_{2\mathrm{RRIstand}}} \right)\cdot\frac{{180}^{0}}{\pi}$$

$${\Delta\theta}_{\mathrm{RRIsupstand}}=\arctan\left( \frac{\alpha_{1\mathrm{RRIsup}}-\alpha_{2\mathrm{RRIsup}}}{1+\alpha_{1\mathrm{RRIsup}}\cdot\alpha_{2\mathrm{RRIsup}}} \right)\cdot\frac{{180}^{0}}{\pi}-\arctan\left( \frac{\alpha_{1\mathrm{RRIstand}}-\alpha_{2\mathrm{RRIstand}}}{1+\alpha_{1\mathrm{RRIstand}}\cdot\alpha_{2\mathrm{RRIstand}}} \right)\cdot\frac{{180}^{0}}{\pi}$$

Analogous derivation follows for changes of angles in RRI signal in supination with slow (0.1 Hz) breathing *Δθ*_RRIsupsup01_, in orthostasis with slow breathing (*Δθ*_RRIstandstand01_), in combined condition *Δθ*_RRIsup01stand01_. Analog principle was applied on DFA regression lines for respiratory signal and changes of angles in respiration signal: under the influence of orthostasis (*Δθ*_Respsupstand_), supination with slow breathing (0.1 Hz) (*Δθ*_Respsupsup01_), standing with slow breathing (*Δθ*_Respstandstand01_) and in combined condition (*Δθ*_Respsup01stand01_).


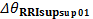

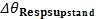

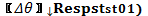


**Angular statistics**

As analyzed in Kalauzi et al., 2012, changes of angles should be estimated by means of formulas for angular mean and standard deviation values.

However, in this research, angular measures were not necessary because samples of *θ_i_* were small and had no aggregating of angle values around critical boundary of ± 180^0^. Thus, usual linear formulas for mean and standard deviation over our sample of n (=20) angles *θ_i_* (*i*=1,…,n, -180^0^< *θ_i_*<180^0^) were applied:

$$mean[\theta i]=\frac{\sum_{i=1}^{n} \theta_{i}}{n}$$

$$std[\theta i]=\sqrt{\frac{\sum_{i=1}^{n} \left( \mathrm{mean}\left[ \theta i \right]-\theta i \right)^{2}}{n}}$$

Appendix III

**Probability estimation for mean and standard deviation of fractal angles**

Changes of mean and standard deviation of inter-fractal angles under influence of body posture and breathing frequency could be observed as elements of graphic presentation of probability distributions of angular values in these specific conditions (Figure 5).

**
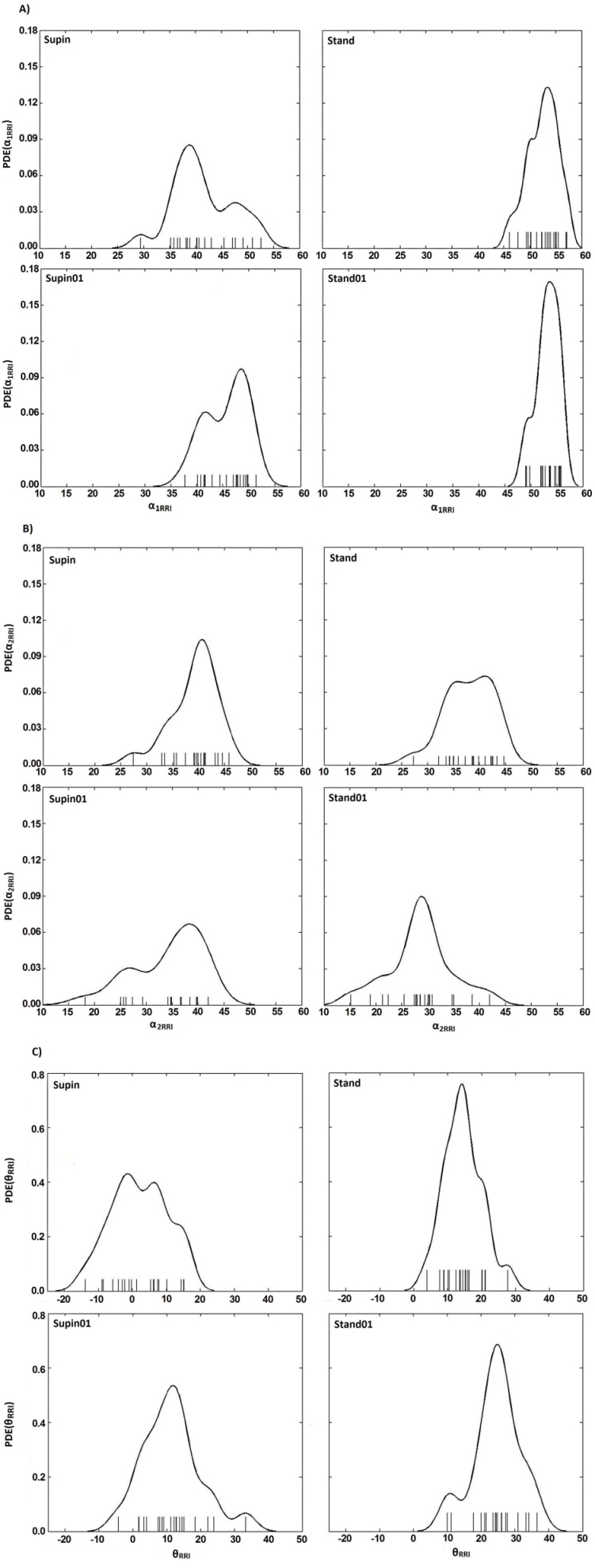
**

**Figure 5.** Changes of distributions of fractal angles for 20 subjects in RRI signal under the influence of breathing frequency and orthostasis: A) α_1RRI_ – short term fractal angle; B) α_2RRI_ – long term fractal angle; C) *θ*_1RRI_ – inter-fractal angle; – supine position ; stand – standing; supin01 – supine position with paced 0.1 Hz breathing; stand01 – standing with paced 0.1 Hz breathing.

**
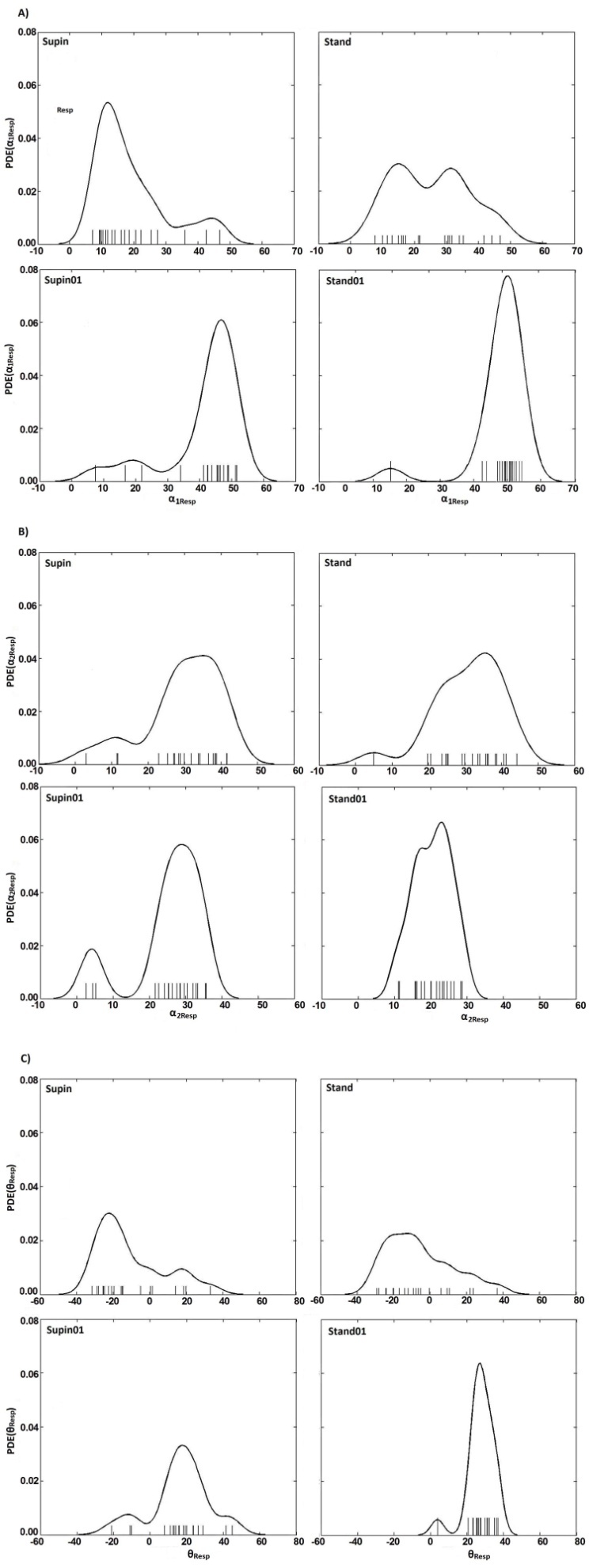
**

**Figure 6.** Changes of distributions of fractal angles for 20 subjects in respiratory signal under influence of breathing frequency and orthostasis: A) α_1resp_ – short term fractal angle; B) α_2resp_ – long term fractal angle; C) *θ*_resp_ – inter-fractal angle; supin – supine position; stand – standing; supin01 – supine position with paced 0.1 Hz breathing; stand01 – standing with paced 0.1 Hz breathing.

Appendix IV

**Application of spectral coherence on evaluation of cardiorespiratory coupling**

Cardio-respiratory coherence is a concept based on usage of spectral coherence in estimation of the degree in which spectral components between RRI and respiration signal are significantly correlated (Daoud et al., 2018). As a function of the frequency it might be expressed by formula:

$$\mathrm{Coh}_{\mathrm{RRI}-\mathrm{Resp}}\left( f \right)=\frac{\left| \mathrm{PSD}_{\mathrm{RRI}-\mathrm{Resp}}\left( f \right) \right|^{2}}{\mathrm{PSD}_{\mathrm{RRI}}\left( f \right)\cdot\mathrm{PSD}_{\mathrm{Resp}}\left( f \right)}$$

where ǀPSD_RRI-Resp_ǀ is magnitude of cross power spectral density of RRI and respiratory signals, PSD_RRI_ and PSD_Resp_ are the power spectral densities of RRI and respiration, respectively.

As described in methods we applied formula for Coh_RRI-Resp_ (f) on each of 20 subjects for 4 conditions they went through. On Figure 7 we can see cardiorespiratory coherence estimated in a representative subject.

| Parameter | supin | stand | supin01 | stand01 |
| --- | --- | --- | --- | --- |
| Breath. frequency [Hz] | 0.3459 | 0.3195 | 0.0983 | 0.0977 |
| Coherence | 0.9483 | 0.897 | 0.9476 | 0.789 |

**
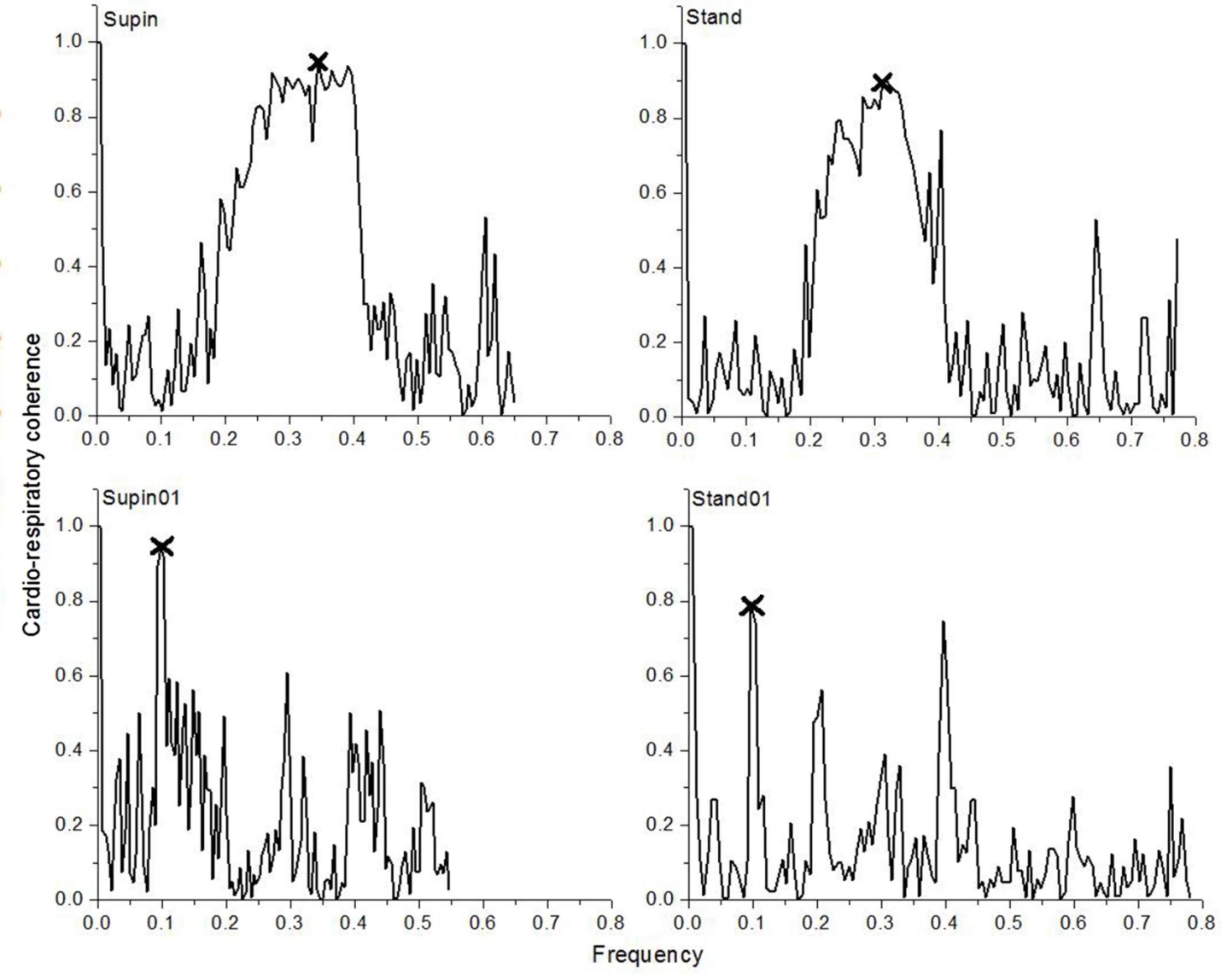
**

**Figure 7.** Graphic representation of cardio-respiratory coherence spectra in a representative subject for 4 conditions: supin – supine position; stand – standing; supin01 – supine position with paced 0.1 Hz breathing; stand01 – standing with paced 0.1 Hz breathing; peaks marked by black "X" marks are maximum values of spectrums (Coh_RRI-Resp_). Table shows x (breathing frequency) and y (coherence) coordinates of these peaks.

While values of Coh_RRI-Resp_ little above 0 reflect high degree of nonlinearity, values close to 1 are accompanied with high degree of linearity (Doud et al., 2018). Thus, we assumed that values higher than 0.8 are signs of strong cardiorespiratory coupling. And as it can be seen from Figure 7 and the inserted table, the highest peaks appear at the frequencies of breathing. We took these peaks to represent linear degree of coupling between RRI and respiration signal.
